# Proteoformics-driven new concept of human growth hormone in clinical endocrinology

**DOI:** 10.3389/fendo.2026.1842422

**Published:** 2026-08-20

**Authors:** Qi Zhang, Yuchen Ma, Ying Liu, Arshad Ali, Yan Wang, Fang Zhang, Liang Chen, Xianquan Zhan

**Affiliations:** 1 Shandong Provincial Key Laboratory of Precision Oncology, Shandong Cancer Hospital and Institute, Shandong First Medical University & Shandong Academy of Medical Sciences, Jinan, China; 2 Shandong Provincial Key Medical and Health Laboratory of Ovarian Cancer Multiomics, &Jinan Key Laboratory of Cancer Multiomics, Shandong First Medical University & Shandong Academy of Medical Sciences, Jinan, China; 3 Department of Medical Imaging, Shandong Provincial Hospital, Shandong First Medical University & Shandong Academy of Medical Sciences, Jinan, China; 4 Laboratory of Cancer Proteoformics, School of Pharmaceutical Sciences, Shandong First Medical University & Shandong Academy of Medical Sciences, Jinan, China

**Keywords:** acromegaly, growth hormone (GH), growth hormone deficiency (GHD), growth hormone proteoforms (GHPs), proteoformics

## Abstract

Growth hormone (GH) is a multifunctional regulator in somatic growth, metabolism, and tissue repair. Its pulsatile secretion is regulated by hypothalamic GH-releasing hormone (GHRH) and somatostatin (SST), and feedback inhibition by insulin-like growth factor 1 (IGF-1). Dysregulation of GH underlies several clinical disorders, including GH deficiency (GHD), acromegaly, and gigantism. The emerging concept of proteoformics has fundamentally reshaped the traditional view of GH: although human GH is encoded by a single gene, it gives rise to multiple proteoforms (GHPs) with distinct biological activities. In-depth study on GHPs pattern benefits understanding its precise molecular mechanism, discovering new therapeutic targets/drugs, and constructing GHPs-based biomarkers. Integration of proteoformics with multi-omics facilitates the construction of dynamic regulatory networks for GH signaling, thereby providing robust support for biomarker discovery and precision medicine strategies in GH-related diseases. Proteoform-based personalized therapy holds promise to advance current treatments such as somatostatin receptor ligands (SRLs), growth hormone receptor (GHR) antagonists, and long-acting GH formulations, with emerging therapies like antisense oligonucleotides and oral GHRH agonists showing preliminary promise. This review examines the potential role of proteoform-centered multi-omics in advancing personalized diagnosis and treatment, discusses challenges in clinical translation, and outlines a roadmap for integrating GH proteoformics into predictive, preventive, and personalized medicine (3PM).

## Graphical Abstract

**Figure:**
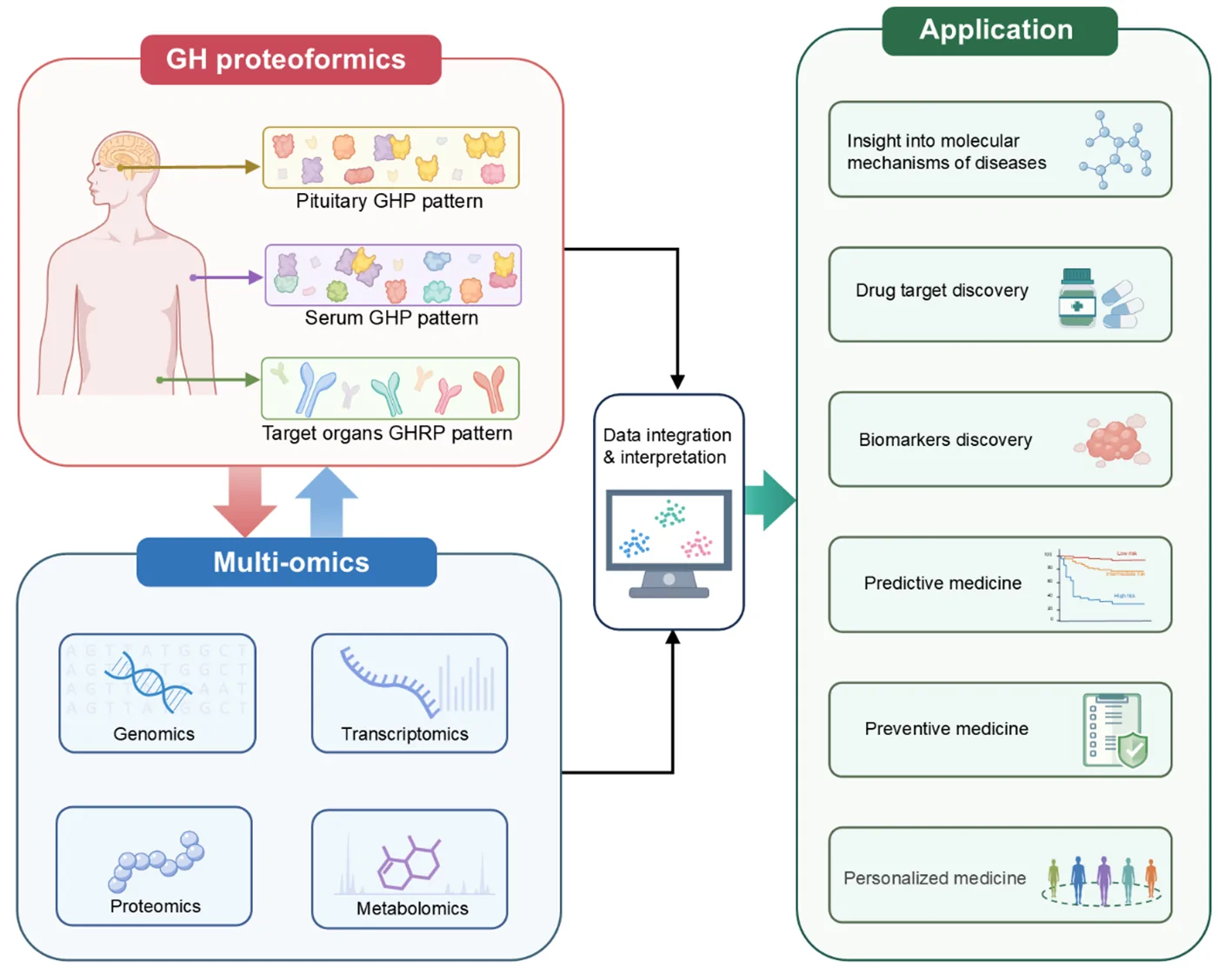
GHP, growth hormone proteoforms; GHRP, growth hormone receptor proteoforms.

## Highlights

- GH proteoforms demonstrate the diversity of GH, a brand-new perspective to reinterpret GH-related disease pathogenesis.
- GH proteoform-centered multiomics represents a promising strategy for precise management of GH-related disease.
- GH proteoform signaling pathway-targeted new-generation drugs represent a promising exploratory direction for GH-related diseases.
- Serum GH proteoform pattern is a promising candidate biomarker for GH-related diseases.

## 1 Physiological function and molecular regulation of growth hormone

### 1.1 The mechanism of GH actions

GH is encoded by the GH1 gene on chromosome 17q23.3 and is a hormone synthesized and secreted in somatotroph cells of the anterior pituitary lobe (1). GH1 in the pituitary gland is subjected to alternative splicing to produce two major mRNA products, encoding a 22 kDa GH molecule with 191 amino acids and a 20 kDa GH molecule with 176 amino acids, respectively (2). However, beyond these two splice variants, human GH exists as a diverse family of proteoforms. These include additional size variants, such as 5 kDa, 17 kDa, and 24 kDa, as well as post-translationally modified forms and proteolytic fragments (1, 3). Each of them has its own biological activities, receptor binding affinities, and metabolic clearance rate. GH is not only a core regulator of body growth and development, but also a key player in metabolism, internal environment homeostasis, immune regulation, and tissue repair (4). Studying only the single “standard” GH molecule overlooks most of its functional diversity, requiring a proteoform-level perspective.

The synthesis and secretion of GH in the anterior lobe of the pituitary gland are dually regulated by hypothalamic GH-releasing hormone (GHRH) and the somatostatin (SST) (5), and are negatively feedback regulated by the insulin-like growth factor 1 (IGF-1) (6). GHRH promotes GH synthesis and release by activating the cAMP-PKA pathway, while SST determines the timing of bursts of GH secretion and antagonizes the action of GHRH. Non-pituitary GH, which belongs to the senescence-associated secretory phenotype, can influence cellular senescence through p53 (7).

**Figure 1:**
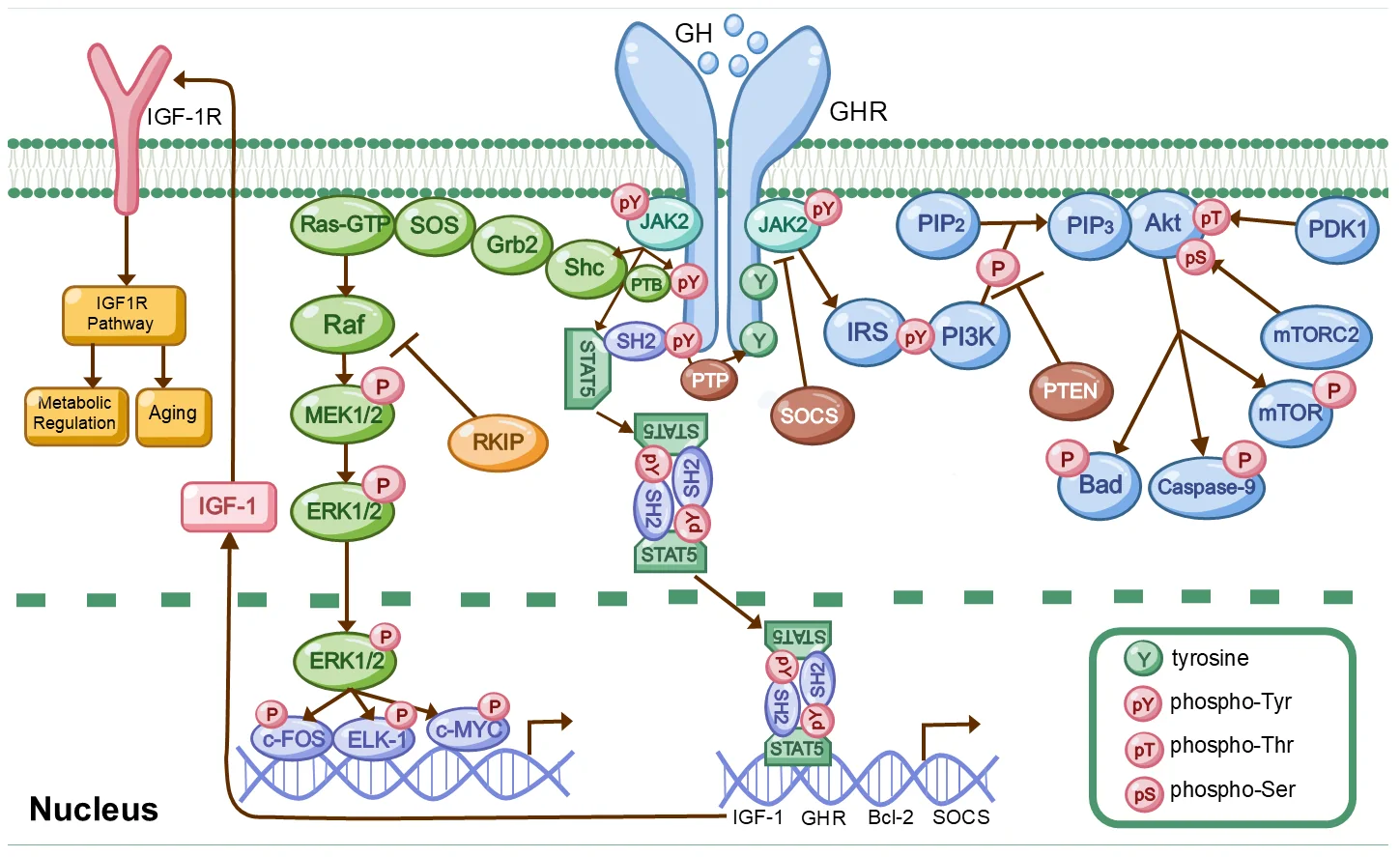
The GH signaling pathway. GH binding to GHR initiates STAT5 phosphorylation and nuclear translocation, and activates the PI3K-AKT and MAPK signaling pathways, leading to a range of biological effects. Akt, Ak strain transforming; Bcl-2, B-cell lymphoma-2; Bad, BCL2-associated agonist of cell death; c-Fos, cellular Fos proto-oncogene; c-Myc, cellular Myc proto-oncogene; Caspase-9, Cysteine-aspartic acid protease 9; Elk-1, ETS transcription factor ELK1; ERK1/2, extracellular regulated kinase 1/2; Grb2, growth factor receptor-bound protein 2; GH, growth hormone; GHR, growth hormone receptor; IGF-1, insulin-like growth factor 1; IGF-1R, insulin-like growth factor 1 receptor; IRS, insulin receptor substrate; JAK2, Janus kinase 2; mTOR, mechanistic target of rapamycin; mTORC2, mechanistic target of rapamycin complex 2; MEK1/2, mitogen-activated protein kinase kinase 1/2; PTEN, phosphatase and tensin homolog; PIP_3_, phosphatidylinositol (3,4,5)-trisphosphate; PIP_2_, phosphatidylinositol (4,5)-bisphosphate; PI3K, phosphoinositide 3-kinase; PDK1, 3-phosphoinositide-dependent protein kinase 1; PTB, phosphotyrosine binding; PTP, protein tyrosine phosphatase; Raf, rapidly accelerated fibrosarcoma; RKIP, Raf kinase inhibitory protein; Ras-GTP, rat sarcoma complexed with guanosine triphosphate; STAT5, signal transducer and activator of transcription 5; SOS, Son of sevenless; Shc, Src homology and collagen; SH2, Src homology 2; SOCS, suppressor of cytokine signaling.

The diversity in the biological functions of GH is due to a complex molecular regulatory network and dynamic secretion pattern. GH initiates signaling by binding to the GH receptor (GHR) on the surface of cell membrane. Notably, distinct GH proteoforms (GHPs) bind to the GHR with different affinities. For instance, the 20 kDa GH displays approximately one-ninth of the GHR binding affinity possessed by the canonical 22 kDa GH (8). GHR is a single transmembrane protein that lacks intrinsic kinase activity in its intracellular domain, but recruits and activates the related tyrosine kinase, Janus kinase 2 (JAK2) (9). Prior to GH binding, GHR exists as dimers, with each GHR monomer binding through its intracellular segment to form a pre-bound complex with one JAK2 (10). Asymmetric binding of GH to the GHR dimer juxtaposes two JAK2 molecules for trans-phosphorylation, ultimately triggering JAK2 signaling activation. Subsequently, JAK2 phosphorylates multiple tyrosine residues in GHR, and the phosphorylated tyrosine in GHR becomes a signal recruitment site, which can bind to Stat5a (signal transducer and activator of transcription 5a), Stat5b and so on (11) (**Figure 1**). The activation of JAK2 triggers the following key events:

1. STAT5 phosphorylation and nuclear translocation: STAT5, recruited by phosphorylated receptors, is phosphorylated by JAK2 to form a dimer, which enters the nucleus through nuclear localization signals, binds to target gene initiators, initiates transcription, and promotes the synthesis of growth-related proteins (12).
2. PI3K-AKT pathway activation: JAK2 phosphorylates the insulin receptor substrate (IRS) to recruit phosphatidylinositol 3-kinase (PI3K), which catalyzes the conversion of phosphatidylinositol (4,5)-bisphosphate (PIP_2_) to phosphatidylinositol (3,4,5)-trisphosphate (PIP_3_). PIP_3_ further activates Ak strain transforming (Akt). Activated Akt further phosphorylates downstream targets and plays vital roles in oxidative stress, cell growth, and apoptosis (13).
3. MAPK pathway regulation: phosphorylated GHR recruits Src homology and collagen (Shc), which is then phosphorylated by JAK2. Subsequently, the growth factor receptor-bound protein 2-son of sevenless (Grb2-SOS) complex is recruited to mediate rat sarcoma (Ras) activation and form the active Ras complexed with guanosine triphosphate (Ras-GTP), Ras-GTP recruits and activates rapidly accelerated fibrosarcoma (Raf), which in turn phosphorylates mitogen-activated protein kinase (MEK) and subsequently extracellular regulated kinase (ERK). The activated ERK translocates to the nucleus, phosphorylates transcription factors, regulates the expressions of genes, and promotes cell proliferation and tissue repair, especially playing a key role in muscle and liver (14).

GH secretion and its systemic regulation are essential for organismal homeostasis. GH deficiency (GHD) is an endocrine and metabolic disorder caused by hypopituitarism. It results in insufficient GH secretion or reduced biological effects. This affects the normal bone development and leads to dwarfism. The biological activity of GH is not only dependent on its total concentration, but also on the specific proteoforms present. An abnormal GHPs pattern could contribute to GHD and other GH-related diseases. GH also stimulates the liver to produce IGF-1, a key mediator that promotes the growth of bones and soft tissues (15). Excessive production of GH leads to two major diseases - acromegaly and gigantism. GH is also associated with several other diseases and treatments. For example, the incidence of non-alcoholic fatty liver disease (NAFLD) is significantly higher in adult GHD (AGHD) patients. The bidirectional regulation of GH on glucose metabolism also links it to diabetes (15). And recent comprehensive review has further highlighted the pleiotropic roles of GH in glucose metabolism, tissue fibrosis, and tumorigenesis, suggesting that its pathophysiological impact extends well beyond what has traditionally been recognized (16).

In addition, the negative regulation of GH is equally important. Negative regulation involves JAK-STAT signaling mediated by PIAS (protein inhibitor of activated STAT), PTPs (protein tyrosine phosphatases), or the SOCS (suppressor of cytokine signaling) (17). When the JAK2-STAT pathway is activated by GH, cells are induced to produce SOCS proteins. These proteins contain a central phosphotyrosine-binding SH2 domain and a conserved C-terminal SOCS box motif (10). The SOCS proteins block signaling and promote receptor degradation by directly binding to either JAK2 or GHR (18). At the same time, GH stimulates the liver to secrete IGF-1. This IGF-1 then feeds back through the circulation to inhibit the release of GHRH from the hypothalamus and reduce pituitary GH synthesis. Phosphatase and tensin homologue (PTEN) down-regulates GH signaling by affecting the interconversion of PIP3 and PIP2, thereby preventing PI3K-AKT pathway activation (19), and PTPs negatively regulate GH signaling. In addition to the two dominant inhibitory modes, tyrosine hydroxylase-expressing neurons provide another key negative feedback loop that automatically regulates GH secretion (20). In conclusion, the concerted regulation of GH levels is essential for normal growth and metabolism and prevents over-secretion-induced disorders.GH directly activates GHR to stimulate the synthesis of IGF-1. IGF-1 is then transported to various tissues through blood circulation and interacts with its main receptor, IGF-1R, to mediate cell survival, growth and proliferation (10). According to previous literature, the growth-promoting effects of GH are mainly mediated through IGF-1. However, recent studies have shown that GH can act directly on target tissues: (i) adipose tissue: GH enhances lipolysis through activation of the β3-adrenergic receptor (β3-AR), a process that does not depend on IGF-1; (ii) muscle tissue: GH acts independently of IGF-1 and directly promotes skeletal muscle cell fusion; (iii) immune system: GH may regulate the migration of immune-related cells through the JAK2-STAT3 pathway to improve immunity; and (iv) central nervous system: GH crosses the blood-brain barrier, promotes synaptic plasticity in hippocampal neurons, and improves cognitive function (21). Because different GHPs have different receptor-binding properties, they may drive these direct effects in a tissue-specific manner. Of note, GH and IGF-1 have opposing effects on metabolic regulation, and this relationship carries important clinical implications for understanding the metabolic disturbances seen in states of GH excess or deficiency (22).

For a long time, GH research focused on total hormone levels and largely ignored functional differences among GHPs. The emerging concept of proteoformics indicates that a single gene can produce multiple protein proteoforms, each with its own job. Looking GH from a proteoform-centered standpoint changes our conventional thinking, as GH forms a diverse molecular family rather than a single uniform hormone. Individual proteoforms differ in receptor-binding affinity, signaling strength, metabolic clearance, and tissue-specific bioactivity. Therefore, disease expressions may arise not only from quantitative alterations in total GH levels, but also from qualitative shifts in the GHPs profile. Distinguishing between these two patterns is important to state the pathogenesis of GH-related disorders, and provides potential directions for developing proteoform-specific biomarkers and targeted therapeutic strategies. Such therapies can selectively suppress pathogenic proteoforms while maintaining the physiological functions of beneficial variants. However, the application of proteoform principles to human disease remains largely exploratory, and most claims of clinical utility are not yet supported by regulatory-grade evidence.

### 1.2 GH pulse secretion

One striking feature of GH secretion is its sex difference. It is mainly characterized by intermittent pulsatile release in males and more stable secretion in females (23). This pulsatile release is mainly regulated by the dynamic balance between GHRH and SST in the hypothalamus. GHRH promotes GH secretion from the pituitary anterior lobe, while SST inhibits it. The two hormones periodically interact to generate intermittent GH pulses. Conventional wisdom suggests that slow-wave sleep (SWS) integrity is closely related to GH secretion. But recent evidence indicates that interrupting SWS may not have a direct or significant effect on GH secretion in adolescent children (24). At the same time, multiple factors influence GH secretion, including age and developmental stage, nutritional status, obesity, metabolism and sex hormones.

This secretion pattern is not only the basis of GH function, but also a key regulatory mechanism for adapting to the body’s energy state. Under prolonged starvation, hypoglycemia and elevated free fatty acids stimulate GH pulse secretion and promote lipolysis, thereby providing energy (25). In the postprandial period, elevated insulin and IGF-1 levels inhibit GH secretion and promote nutrient storage through different signaling pathways (26). During exercise stress, intensity above a threshold triggers GH pulses through relevant signals, leading to a significant increase in hGH secretion to support energy mobilization and tissue repair (27). One more layer of complexity is that different GHPs have different metabolic clearance rates. That means they don’t hang around in the blood for the same amount of time. Therefore, the exact mix of proteoforms can change the amplitude and duration of GH pulses, making the link between secretion pattern and biological function even messier.

Over the last few years, exploratory research has been conducted in various areas to develop treatment strategies targeting the pulsatile secretion of GH. The synthetic GHRH analogs mimic the physiological pulsatile pattern by permanently binding to endogenous albumin to prolong drug half-life. This reduces injection frequency and elevates basal GH levels while maintaining intact pulsatile GH secretion (25). Gene therapy has been explored to restore physiological GH secretion using AAV vectors or stem cell technology, but it remains experimental (28). A newly developed GH transdermal microneedle patch can mimic the release pattern through biological rhythms, thereby maximizing drug utilization (29).

### 1.3 Effects of GHPs and modifications on GH functions

**Figure 2:**
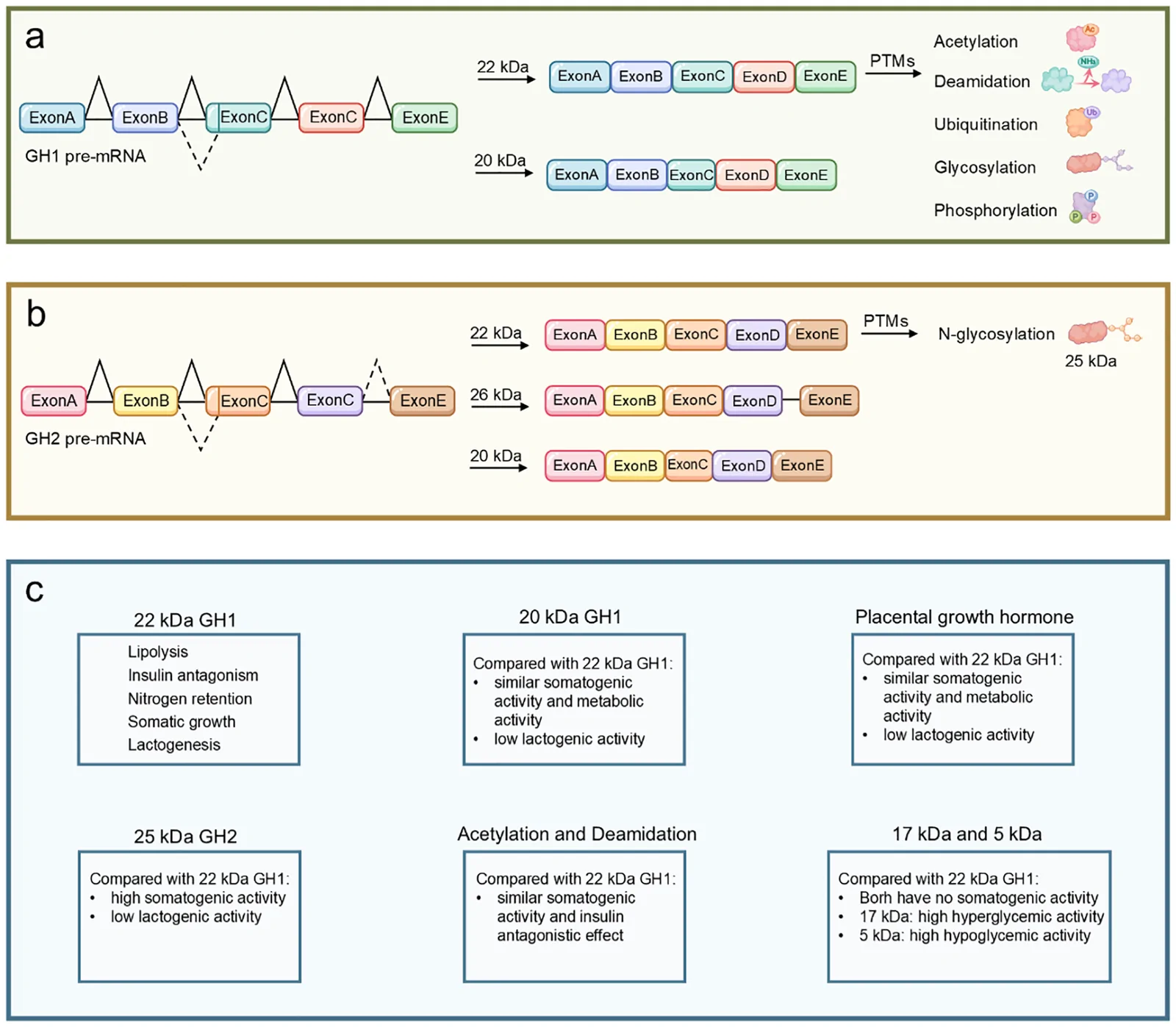
Generation and functions of major growth hormone proteoforms. **(a)** Alternative splicing of GH1 pre-mRNA yields 20 kDa GH and 22 kDa GH; extensive proteoforms are further derived from post-translational modifications (PTMs) of the 22 kDa GH. **(b)** Alternative splicing of GH2 pre-mRNA yields 22 kDa, 26 kDa and trace amounts of 20 kDa GH products. N-glycosylation at residue Asn140 of the 22 kDa GH produces the 25 kDa GH. **(c)** This section summarizes the primary physiological functions of GH proteoforms. The proteoforms encoded by the GH2 gene are also collectively referred to as placental growth hormone (PGH). GH: growth hormone. PTMs: post-translational modifications.

hGH exists as multiple proteoforms, including 5 kDa, 12 kDa, 17 kDa, 20 kDa, 22 kDa, 24 kDa, 25 kDa, and 26 kDa (**Figure 2**). This heterogeneity arises from genetic factors, mRNA processing, and post-translational modifications (PTMs). The hGH gene family comprises five closely related genes: GH-N (GH1), chorionic somatomammotropin hormone like 1 (CS-L), chorionic somatomammotropin hormone A (CS-A), GH-V (GH2), and chorionic somatomammotropin hormone B (CS-B) (30). GH-N is expressed exclusively in the pituitary gland, whereas the other four genes are expressed in the placenta (31). CS-A and CS-B encode the same product, known as chorionic somatomammotropin or placental lactogen. CS-L encodes a CS-like protein that is likely non-functional.

During transcription of the GH-N gene, alternative mRNA splicing produces the major 22 kDa and minor 20 kDa products. The 22 kDa form is the most abundant primary GHP, containing 191 amino acids with two disulfide bonds. The 20 kDa hGH is composed of 176 amino acids and is the second most abundant form, accounting for approximately 5%-10% of pituitary GH. It tends to dimerize and has a slower metabolic clearance rate than the 22 kDa form (3). The 20 kDa and 22 kDa hGH forms have roughly equal growth-promoting activity. However, the 20 kDa hGH has approximately one-ninth and one-third the binding affinity for the GHR and prolactin receptor (PRL-R), respectively, compared to the 22 kDa hGH (8). This difference is due to their interactions with distinct receptors.

The GH-V gene produces four products: 22 kDa, 20 kDa, 25 kDa, and 26 kDa. The 22 kDa, 20 kDa and 26 kDa forms result from alternative mRNA splicing, with the 22 kDa form being the major one. This differs from pituitary GH by 13 amino acids. The 25 kDa proteoform arises from N-glycosylation at asparagine 140 (Asn140) of the 22 kDa, it exhibits high growth-promoting activity yet low lactogenic activity compared to pituitary GH.

Placental growth hormone (pGH) is a product of the GH-V gene, synthesized and secreted by placental syncytiotrophoblast cells. From 12 to 20 weeks of gestation until full term, pGH in maternal circulation gradually replaces pituitary GH. pGH is involved in regulating maternal metabolism during pregnancy. It is secreted in a non-pulsatile manner, with concentrations rising steadily as pregnancy progresses and dropping rapidly within one hour after delivery. pGH is secreted only into the maternal circulation and is not detected in fetal blood. pGH binds to GHR with an affinity similar to that of pituitary GH, but it has much lower affinity for the prolactin receptor. This gives it high growth-promoting activity but low lactogenic activity (32).

The 24 kDa hGH is an O-glycosylated proteoform of the 22 kDa GH-N gene product at threonine 60 (Thr60). The oligosaccharide at Thr60 localizes high-affinity binding site 1 of hGH. It may affect receptor binding affinity and post-binding conformational changes through steric hindrance, thereby influencing biological activity. The 24 kDa hGH may have hGH antagonist properties (33). The 5 kDa and 17 kDa forms are present in pituitary extracts and the bloodstream. The specific enzyme responsible for this breakdown in humans has not yet been identified, although treatment of 22 kDa hGH with thermolysin *in vitro* produces 5 kDa and 17 kDa fragments (34). The 17 kDa hGH has significantly enhanced diabetogenic activity compared to the 22 kDa form. This activity is separate from its growth-promoting and insulin-like properties. The 5 kDa fragment exhibits a greater insulin potentiating activity than 22 kDa GH1. In immunoassays, the 17 kDa fragment cross-reacts with certain anti-GH antibodies, interfering with GH immunoassay results, while the 5 kDa fragment does not cause such interference (34). A 12 kDa N-glycosylated form is also present in human pituitary extracts. It may derive from GH-V gene expression in the pituitary or from other unknown GH-related genes (35).

**Figure 3:**
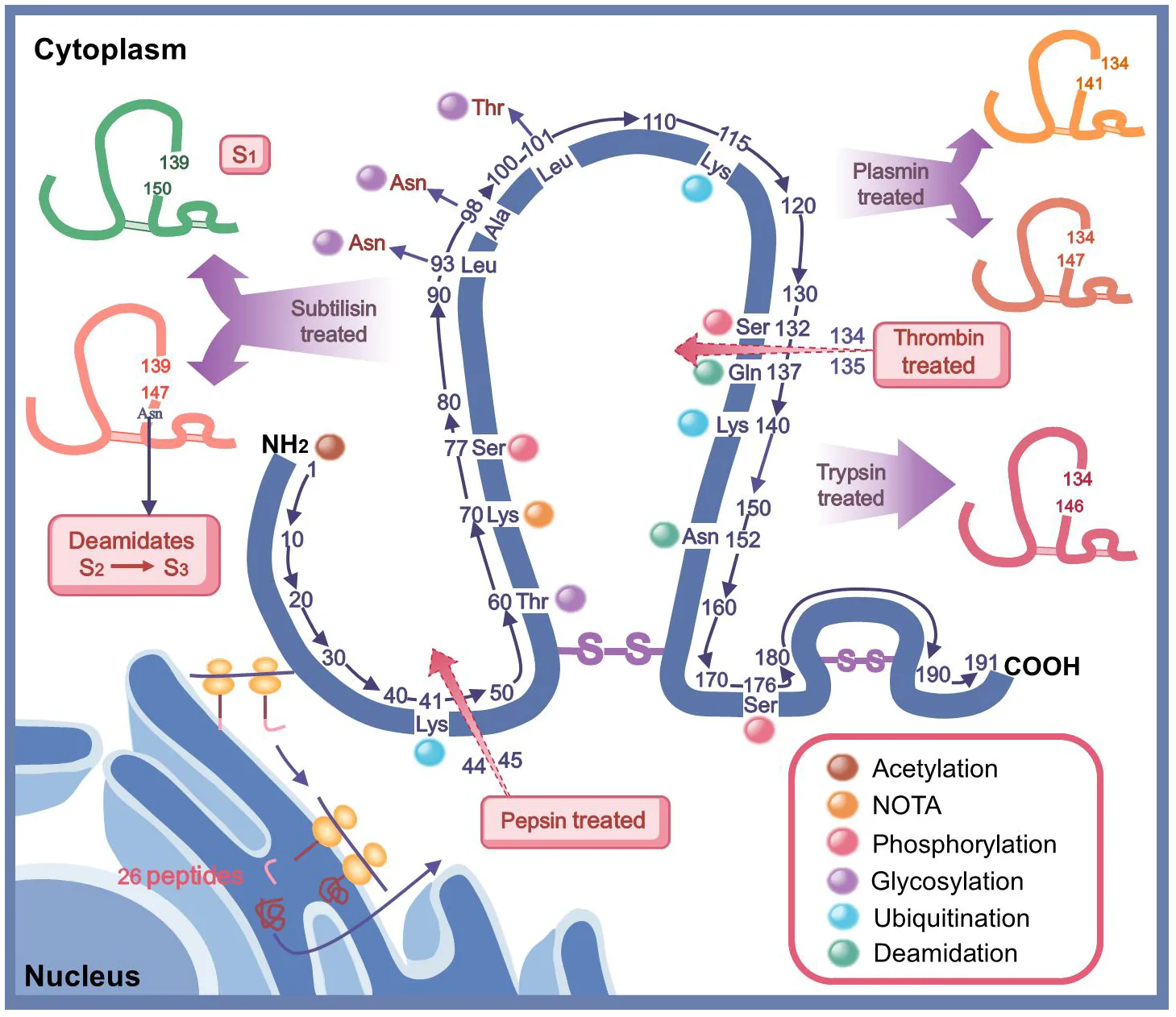
The modification of the GH product at the protein level. After the 26-amino-acid signal peptide is cleaved from the GH precursor in the endoplasmic reticulum, the protein undergoes a variety of structural modifications. The image illustrates specific sites of multiple post-translational modifications, including acetylation, phosphorylation, glycosylation, ubiquitination, and deamidation. GH can also be acted upon by a range of enzymes such as subtilisin, pepsin, plasmin, thrombin and trypsin. The cleavage of GH by these enzymes generates distinct proteoforms. Ala, alanine; Asn, asparagine; Gln, glutamine; Leu, leucine; Lys, lysine; NOTA, 1,4,7-triazacyclononane-1,4,7-triacetic acid; Ser, serine; Thr, threonine.

PTMs are crucial for protein function. GH PTMs include phosphorylation, deamidation, acetylation, glycosylation, ubiquitination, oligomerization, and proteolysis (**Figure 3**). Initially, GH is synthesized as a precursor with a 26-peptide N-terminal sequence, which is cleaved in the endoplasmic reticulum. Our team identified phosphorylation at serine77 (Ser77), Ser132, and Ser176 in GH (2). Phosphorylation of GH plays a central role in signal transduction, and its function relies on a tightly regulated phosphorylation-dephosphorylation cycle in cells. Deamidation at glutamine137 (Gln137) and Asn152 changes the cleavage sites of GH by subtilisin. Notably, hGH Glu137 is more resistant to subtilisin hydrolysis than the wild-type. Acetylation at the N-terminus of GH produces “Fast-hGH”, which has a faster electrophoretic mobility than the main component at pH 7.8 (36).

GH variants with N-glycosylation were constructed by replacing leucine93 (Leu93), alanine98 (Ala98), and leucine101 (Leu101) with asparagine or threonine. The study found that the degree of sialylation of the sugar chains was positively correlated with the *in vivo* half-life of GH (35). Three potential ubiquitination sites (K67, K141, and K166) were identified in the hGH precursor with ubiquitination site prediction programs. Mutation of residue K141 to arginine significantly reduced ubiquitination in the blood and prolonged GH’s half-life (37). Introducing the chelating agent 1,4,7-triazacyclononane-1,4,7-triacetic acid (NOTA) at lysine 70 (Lys70) in GH allows it to serve as a targeted radiopharmaceutical carrier without interfering with GHR binding. This provides a new approach for diagnosing and treating cancers with high hGHR expression and expanding GH’s applications in cancer diagnosis and treatment.

The proportion of GHPs is also related to the development of certain diseases. One study found that in some short children with non-growth hormone deficiency (non-GHD), the proportion of non-22 kDa GHPs in the circulation increases (38). Also, compared to healthy individuals, untreated acromegaly patients have an increased proportion of non-22 kDa GH subtypes relative to total GH. This proportion is positively correlated with tumor size, 24-hour average GH concentration, and serum prolactin levels (39). These proteoforms have different immunoreactivities and biological activities. Some monoclonal assays lack reactivity toward the 20 kDa GH isoform, so this analytical limitation should be fully considered when selecting detection methods. Such immunoassays underestimate the total concentration of biologically active GH in circulation. This measurement bias interferes with the interpretation of GH stimulation test results in short children screened for growth hormone deficiency, bringing potential risks of misdiagnosis and unnecessary clinical intervention (40). Therefore, GH heterogeneity, GHP, should be considered in the diagnosis and treatment evaluation of GH-related diseases such as GHD and acromegaly (3, 36). However, the precise functional consequences of altered proteoform ratios, and how they mechanistically contribute to pathology rather than merely correlate with it, require more rigorous investigation.

### 1.4 GHPs and GHRPs

After secretion from the pituitary, GHPs enter the bloodstream and circulate throughout the whole body. GHPs exert physiological regulatory functions by specifically binding to GHR on target organs and target cells, which subsequently activates downstream GH signaling cascades. Of course, GHR also undergoes alternative splicing and diverse PTMs to produce multiple GHR proteoforms (GHRPs). Comprehensive understanding of this ligand-receptor proteoform heterogeneity is fundamental to the present study.

#### 1.4.1 GHPs and modifications in patient sera in GH-related diseases

Among GH-related disorders, serum GHPs and their modifications are a key research area. GH is released from the anterior pituitary and enters the bloodstream. Under normal physiological conditions, GH in the serum exists in many different isoforms. Genetically speaking, the hGH-N gene is expressed in pituitary, and its translation products form two main isoforms: 20 kDa and 22 kDa (1). The 22 kDa GH makes up about 75% of the GH secreted by the pituitary and is the main biologically active component, while the 20 kDa isoform accounts for roughly 10% (2). Besides these, pituitary also produces some other forms of GHPs. These GHPs keep specific proportions in the serum and work together to maintain the body’s normal growth and metabolism (1).

Serum GHPs and their modifications are markedly altered in GH-related diseases. In diseases like acromegaly the changes in serum GHPs are very complex. PitNETs are a common cause of excessive GH secretion. In patients with such adenomas, not only is the total level of GH in the serum much higher, but the levels of specific GHPs are also different from those in normal people (36). Studies have found that there are abnormal expressions of many GHPs in GH-secreting tumors, including proteins involved in mRNA splicing and protein export. The expression of some proteins is also related to changes in GH levels, and tumor cells at different stages of differentiation may express different proteins such as GH1 and GH2. These may provide clues for understanding the proteoforms and modifications of GHPs in the serum (41).

Furthermore, GHPs with different modifications in the serum may play different roles in the progression of the disease, and some modified GHPs may serve as potential early biomarkers for the disease. Monitoring how serum GHPs and their modifications change during treatment may provide valuable insights for evaluating treatment efficacy. For example, after surgical removal of PitNETs or drug treatment in acromegaly patients, changes in the levels of total serum GHPs and related growth factors in the GHP pathway can indicate how well the treatment is working (42).

Serum testing has the advantages of being cost-effective, minimally invasive, and highly accurate. Therefore, serum GHPs represent promising candidate biomarkers, but their sensitivity, specificity, and predictive value require rigorous evaluation in multicenter prospective studies.

#### 1.4.2 GHRPs and modifications in GH target organ in GH-related disease

In target organs, GHR exists as multiple proteoforms (GHRPs). These proteoforms and their associated modifications may play a critical regulatory role in GH-related disorders. GHR belongs to the cytokine receptor superfamily. It is a 70-kDa protein composed of 620 amino acids, with an extracellular ligand-binding domain, a single transmembrane domain, and a cytoplasmic component. It is highly expressed in tissues such as the liver, fat, muscle, and kidneys (43). GHR mainly exists as monomers on the cell membrane. When GH binds to two different sites in the extracellular domain of GHR, it triggers receptor dimerization, which in turn induces a series of signal transduction events (44). Under normal circumstances, this precise signal transduction mechanism maintains the stable operation of physiological processes such as body growth and metabolism.

In GH-related diseases such as GHD and acromegaly, the GHRPs and their modification status in target organs undergo significant changes. In GHD patients, in addition to insufficient GH secretion, the GHR in target organs may also be abnormal. Some GHD patients have GHR gene mutations, most of which occur in the extracellular ligand-binding domain, leading to GHR dysfunction or GH insensitivity (GHI). GHR mutations in GHD can cause misfolding and impaired membrane trafficking, reducing functional GHR and blocking signaling. In acromegaly, chronic GH excess alters GHRPs expression and modifications, potentially driving sustained pathway activation (45). By intervening in the abnormal expression or modification of GHRPs, it is expected to regulate the GH signal pathway, bringing new breakthroughs in the treatment of GH-related diseases.

#### 1.4.3 Exploration of the specific binding between serum GHPs and GHRPs in GH-related diseases

In disease states, the physiological specific binding between GHPs and GHRPs is markedly altered. In GHD, for instance, patients not only have insufficient GH secretion, but also have abnormal types and levels of serum GHPs, which inevitably affects their binding to GHRPs. GHR gene mutations in the extracellular domain can produce structurally abnormal GHRPs that can’t effectively bind normal GHPs, or form low-activity complexes, leading to severe blockage of GH signal transmission and growth retardation (46).

Research into the binding mechanisms between GHPs and GHRPs is increasingly becoming a key breakthrough in driving precision advancements in related diseases and fields. Its value runs through the entire process of disease prevention, diagnosis, and treatment, and establishes a deep connection between molecular mechanisms and clinical translation. From a preventive perspective, research on the binding mechanism of GHPs and GHRPs provides a molecular basis for early intervention in disease risks. For aging-related diseases, regulating GHR levels may maintain metabolic function while delaying aging; in populations with high d3-GHR expression, the risk of vertebral fractures (VFs) increases, and regular monitoring can enable early prevention and intervention of the disease (47). In the field of diagnosis, such research will break the traditional “one-size-fits-all” diagnostic model and move towards precise stratification based on “isoform fingerprints”. In terms of treatment, related research will also promote the innovation from “broad-spectrum intervention” to “isoform targeting”.

Of course, the development of this field still faces technical challenges: single-cell multiomics technology needs to further improve resolution to analyze tissue-specific proteoform profiles (48); off-target risks of gene editing need to be avoided through high-fidelity tools (49); and the reduction of individual detection costs depends on the popularization of high-throughput sequencing technology (50). However, it is foreseeable that as these bottlenecks are overcome, research on GHPs/GHRPs will drive a shift in the management of endocrine disorders from “symptom control” to “mechanism-based interventions,” and to pave the way for the precise prediction, classification, and targeted regulation of these diseases.

## 2 From diagnostic deficits to proteoform precision

GH-related disorders are consistently difficult to diagnose accurately at all clinical stages, including early symptom recognition, laboratory verification and final result interpretation. These widespread limitations often lead to years of delayed diagnosis and reduce the reliability of disease classification, treatment planning and prognostic judgment. To overcome these clinical deficiencies, we propose establishing a standardized GHPs reference profile towards an improved diagnostic framework, which is expected to resolve current diagnostic obstacles and facilitate the shift toward 3PM.

### 2.1 Systemic challenges from clinical recognition to laboratory detection

Diagnostic delay stands as one of the most prevalent clinical challenges, particularly evident in patients with acromegaly. Epidemiological observations indicate that the diagnostic latency of acromegaly commonly ranges from 6 to 10 years, and this unsatisfactory situation has not been substantially improved over the past decades (51, 52). Such prolonged delay is primarily attributed to the non-specific early manifestations of the disease. Early-stage patients predominantly present with generalized symptoms including sleep apnea syndrome, arthralgia and metabolic disorders, whereas typical characteristic signs emerge at a relatively late disease stage. Consequently, most patients have developed irreversible organic damage to multiple vital organs (53).

The inherent technical limitations of GH stimulation tests constitute a major bottleneck in the diagnosis of GHD. Although GH stimulation tests are currently regarded as the gold standard for GHD diagnosis, this canonical diagnostic tool has notable methodological flaws. Accumulated clinical studies have verified poor repeatability of GH stimulation test results. Multiple patient-related factors, such as age, gender, pubertal status, nutritional condition and body weight, can profoundly affect GH secretory responses. In addition, there is no unified cut-off value for clinical diagnosis, and significant discrepancies exist between different stimulation protocols as well as in repeated tests using the same protocol, resulting in unstable and inconsistent detection outcomes (54, 55).

Heterogeneous methodologies for GH immunoassay represent a persistent technical challenge in clinical endocrinology. As early as 1998, relevant laboratory quality assessment studies demonstrated that GH immunoassays exhibited the poorest inter-laboratory consistency among all hormone detection items. For identical serum samples, GH concentrations measured by different laboratories using distinct assay methods could vary by two to three times (56, 57). The fundamental mechanism underlying this discrepancy lies in the fact that different detection antibodies target diverse epitopes of heterogeneous GH isoforms in human serum (58). To date, novel detection techniques designed to resolve this issue are still in the experimental stage and have not been clinically implemented (59).

Discordance between serum insulin-like IGF-1 and GH levels further complicates clinical decision-making. Serum IGF-1, which reflects integrated systemic GH secretion, has been widely adopted as a first-line screening biomarker for acromegaly. Nevertheless, inconsistent results between GH and IGF-1 measurements are frequently encountered in routine clinical practice (60, 61). This phenomenon can occur in early disease stages or under suboptimal treatment status, and may also stem from pre-analytical errors, technical detection defects, physiological fluctuations and concomitant pathological conditions. Such biomarker discordance precludes accurate disease evaluation relying on a single indicator, necessitating comprehensive multi-dimensional clinical judgment and substantially increasing the complexity of clinical diagnosis.

Moreover, conventional clinical assays fail to distinguish individual GHPs, leading to incomplete molecular profiling in disease assessment. Endogenous GH exists as a heterogeneous protein family consisting of multiple isoforms rather than a single molecular entity. However, traditional immunoassays only measure total serum GH concentration without differentiating distinct GHPs. This generic detection approach masks dynamic alterations in the proportion of different GH isoforms under varying physiological and pathological conditions. Accordingly, current diagnostic and therapeutic evaluations are based on incomplete molecular information, leaving inherent deficiencies in diagnostic precision (58).

Overall, the current diagnostic system for GH-related diseases has prominent deficiencies across key procedures, including clinical symptom identification, functional stimulation testing, laboratory immunoassay, biomarker interpretation and molecular characteristic analysis. There is an urgent need to renovate the traditional diagnostic framework and establish a standardized, precise diagnostic system for GH-associated diseases.

### 2.2 Toward a standardized and precision-based diagnostic system

Against the aforementioned diagnostic dilemmas, constructing a disease diagnostic reference atlas based on GHPs represents a fundamental paradigm shift, which breaks through the bottlenecks of conventional diagnosis at the molecular level and delivers multiple clinical advantages.

First, it enables precise molecular subtyping of GH-related diseases. The profiling of GHPs undergoes dynamic and specific changes under different physiological and pathological conditions. High-resolution mass spectrometry and other advanced technologies support systematic characterization of disease-specific GHPs fingerprints for various GH-associated disorders. Previous research has identified 46 distinct GHPs in GH-secreting PitNET tissues, while only 35 proteoforms are detected in normal pituitary tissues. The 11 differential proteoforms discovered might have the potential to serve as disease-specific molecular biomarkers (36). By matching individual patients’ GHPs profiles with the standardized reference atlas, the traditional rough diagnostic mode based merely on GH concentration levels can be upgraded to precise molecular typing grounded on unique proteoform fingerprints.

Second, this approach facilitates early predictive diagnosis and improves overall diagnostic accuracy. Variations in the proportion of specific GHPs occur far earlier than abnormal fluctuations in total GH concentration, serving as an early indicator of impaired GH secretory function (8, 58). The establishment of a standardized GHPs reference atlas allows early disease warning and predictive diagnosis by capturing subtle dynamic changes in serum GHPs composition. Replacing total GH concentration with functional proteoform profiling as the core diagnostic indicator is expected to enhance the biological relevance and clinical accuracy of disease diagnosis.

Third, it guides individualized therapeutic strategies and advances disease management into the 3PM era. Distinct GHPs signatures are correlated with heterogeneous disease biological behaviors and variable treatment responses (62). Comparative analysis of patients’ personal proteoform profiles and the standardized disease reference atlas enables accurate prediction of therapeutic sensitivity, precise patient stratification and individualized treatment regimen optimization. Dynamic monitoring of proteoform spectrum changes during treatment also supports real-time efficacy evaluation and prognostic prediction (1). This innovative diagnostic paradigm transforms GH disease management from passive symptomatic intervention to proactive molecular risk prediction, and from generalized population-based treatment to refined individualized intervention, ultimately realizing the upgrade from symptom-driven diagnosis to molecular mechanism-driven precise management.

## 3 Technological innovations in GH proteoformics

Proteoformics (63, 64), as a systematic study of the expression, modifications, interaction, and function of a proteoform (65–68), includes two-dimensional gel electrophoresis (2-DE), liquid chromatography (LC), mass spectrometry (MS), protein microarrays, antibody/antigen arrays, reverse phase protein arrays (RPPA), proximity extension assay (PEA), and other core technologies (69, 70). The integrated analytical workflow for protein subtypes (proteoforms) has become an important research tool in the field of GH and GH-related disorders (36, 59). By comparing the protein expression and proteoform profiles of GH in normal tissues and disease states, we aim to reveal the molecular details of the GHP signaling pathway, construct dynamic regulatory network models centered on proteoforms, and focus on analyzing disease-related network remodeling (36, 71), and identify patient-specific drug targets or drugs for precision personalized drug design, delivery and therapy (72–74).

Traditional immunoassays suffer from cross-reactivity issues when distinguishing protein forms. In contrast, a targeted LC-MS/MS strategy enables specific detection and differentiation of the two major GH isoforms, 20 kDa and 22 kDa, in human plasma by identifying their characteristic peptide signatures. This overcomes the inherent limitations of immunological methods, achieving high sensitivity and specificity in identifying GHPs (75). The detection approach highlights the unique advantages of proteoform analysis in resolving heterogeneous GH subtypes and drives ongoing technological advancements in the study of GHPs.

Now, different proteoformics technologies are at distinct stages of clinical readiness for GH-related diseases. Mass spectrometry-based Orbitrap Astral technology provides ultra-high scanning speed and high sensitivity for complex sample analysis, enabling the detection of subtle abnormal signs (76). This technical advantage lays a foundation for establishing the proteoform profiling system of GH-related diseases and has potential to improve the comprehensiveness and accuracy of future clinical diagnosis. The 4D-diaXLMS uses a four-dimensional data-independent acquisition method for cross-linking proteome analysis, achieving accurate quantification and high coverage in complex matrices (77). Down the road, this technology is expected to analyze abnormal proteoform interaction networks in GH resistance, pinpointing abnormal binding sites between GHR and signaling proteoforms. It may provide a molecular basis for etiological diagnosis and offer new insights for targeted treatment strategies.

In the field of trace proteoform detection, the Olink PEA technology, based on the principle of dual antibody recognition and DNA-encoded signal amplification, can achieve ultra-low abundance proteoform detection at the fg/mL level (78). In pediatric GHD, where serum levels of GH/GHPs are extremely low and conventional assays yield false negatives. If the Olink PEA technology can stably detect the differential expression of these trace proteoforms, it will provide a reliable tool for the early screening and prognosis assessment of GHD.

​Furthermore, spatial proteoformics represented by CODEX and MIBI enables *in-situ* visualization and spatial distribution analysis of tissue proteoforms via multi-antibody labeling and imaging mass spectrometry (79). If these techniques can be applied in the diagnosis of GH-secreting pituitary neuroendocrine tumors (PitNETs), the spatial distribution characteristics of proteins related to GH synthesis in the tumor tissues might be clearly presented. This could help pathologists accurately determine the invasion range and differentiation degree of the tumor and provide important references for the formulation of surgical plans.

The integration of artificial intelligence (AI) technologies further expands the application boundaries of proteoformics in GH-related disease diagnosis. For instance, AlphaFold3 and D-l-TASSER can predict three-dimensional structures from proteoform amino acid sequences (63). The AI-driven automated preprocessing system optimizes sample preparation process through machine learning, shortening detection time and improving the experimental reproducibility. This is crucial for the standardization of sample detection in clinical diagnosis and could effectively avoid diagnostic errors caused by operational differences (80). Most AI applications in this field remain in the exploratory stage and lack large-scale clinical validation. The greatest potential of AI in this field likely lies in multi-dimensional data integration and prediction. Using large-scale GH-related proteoformics data from PRoteomics IDEntifications (PRIDE) (https://www.ebi.ac.uk/pride/) (81) combined with AI prediction models [such as deep learning models like Proteomizer (82) and BBATProt (83)], it may be possible to construct a diagnostic prediction model for GH-related diseases. Notably, recent advances have demonstrated the value of AI in endocrinology: deep learning algorithms applied to facial photographs can improve the early diagnosis of acromegaly by recognizing subtle phenotypic features that often escape conventional clinical assessment, but this model generalizability across different populations remains unproven (84). This successful paradigm provides a blueprint for developing GH disease prediction models that integrate large-scale GHPs data, clinical measures, and imaging findings for earlier, more accurate intervention. As a concrete example, machine learning has been used to build a predictive model for resistance to first-generation somatostatin receptor ligands (SRLs) in patients with acromegaly. By integrating analyses of multiple biomarkers, this approach holds promise for more precise patient stratification before treatment (85).

Additionally, Spatial and Cell-Type Proteomics (SCPro) combines the ultra-high sensitivity of microfluidics and iPAC technology, enabling quantitative analysis of proteins or even proteoforms at the single-cell level (86). The same AI-driven approach might be extended to construct diagnostic prediction models for broader GH-related diseases by combining large-scale proteoformics data, clinical measures, and imaging. Notably, these technologies must now be adapted to distinguish specific GHPs, not just total protein levels, to fully exploit the proteoform-centric paradigm. It is worth saying that the collaborative development of these technologies is not a simple “technological stacking”, but has formed a closed loop from “molecular mechanism analysis to clinical diagnostic application”. While mass spectrometry-based proteoform analysis is advancing rapidly, routine clinical development is hampered by issue of standardization, throughput, and data interpretation (87, 88). The development of these technologies represents a promising technical foundation that may facilitate the future transition from “empirical medicine” to “precision medicine” in the diagnosis of GH -related diseases, although their routine clinical deployment remains hindered by standardization, throughput, and data interpretation challenges.

## 4 Proteoform-centered multiomics-driven personalized medicine in GH-related diseases

With the rapid development of precision medicine, personalized medicine has great potential in the diagnosis and treatment of GH-related diseases, whose secretion, metabolism, and physiological functions are precisely regulated by genetic, epigenetic, proteomic, and metabolomic factors (89). The integration of multiomics provides the “raw materials” for constructing molecular models and offers new perspectives for elucidating individual variations, promising to advance in-depth research on GH-related disorders and transform clinical practice (90). For these approaches to realize their potential, a proteoform-aware integration framework is required, where genetic, transcriptomic, and metablomic variants are explicitly linked to the resulting protein species (71). In the reality, all these genetic, transcriptomics, and metabolomic variants, and protein species (proteoforms) function in a dynamic pathway network system in direct and indirect interaction manners in GH-related diseases (63).

### 4.1 Associations of genomic variations with GHPs landscape changes in GH-related diseases

Genomic testing has become a key tool for accurate diagnosis of difficult diseases, and trio-whole exome sequencing (Trio-WES) plays an important role in identifying the genetic etiology of syndromic dwarfism in patients with unknown etiology (91). For children born younger than gestational age who have not achieved catch-up growth and whose etiology cannot be clarified by conventional diagnosis, in-depth genomic analysis can reveal potential (epigenomic) genomic alterations, which can provide important clues for the diagnosis and treatment of the disease (92).

In the diagnosis of GHD, WES can effectively compensate for the limitations of false-negative clinical diagnosis. Rare variants in the LHX3, SUFU, POLR3A, and CREB3L4 genes were found to be significantly enriched in GHD patients, suggesting that these may be new pathogenic genes associated with GHD (93). These genomic variants may induce specific GH proteoform shifts, yet the direct causal chain from mutation to altered proteoform pattern remains largely unexplored. In addition, the finding that some patients with clinical manifestations consistent with isolated GH deficiency type IA (IGHD IA) are not caused by deletions or mutations in the GH1 gene as traditionally recognized, but rather by compound heterozygous mutations in the GH1 gene (splicing mutations originating from the mother and 22 kb deletion mutations in the father) has deepened the understanding of the genotype-phenotype association in IGHD IA (94).

Genomic testing also has great potential for risk prediction and individualized management. The pathophysiological mechanism between the activation of the cAMP signaling pathway and DNA damage and genomic instability in GH-secreting PitNETs was clarified by using WES and other methods, providing a new basis for the precise classification and targeted treatment of GH-secreting PitNETs (95). Meanwhile, studies have demonstrated that most GH-secreting PitNETs have mutations in the GNAS gene (96). It is noteworthy that GNAS mutations can potentially affect the proteoform landscape of GHR and downstream signaling proteins, an area that warrants systematic investigation. In addition, mouse studies have shown that abnormalities in the Tmem263 gene can lead to growth retardation, dwarfism, and impaired skeletal development, suggesting that rare mutations or variants in the human Tmem263 gene may be associated with Laron syndrome-like dwarfism and GH insensitivity, and are expected to be a potential target for the screening of children with idiopathic growth disorders (97). Therefore, linking specific genomic variants to alterations in the GHPs landscape is a necessary step to understand how genetic lesions translate into functional changes in GH-related diseases.

### 4.2 Associations of transcriptomic variations with GHPs landscape changes in GH-related diseases

Transcriptomics studies help to deeply analyze the molecular mechanisms of disease development. Transcriptomics analysis of subcutaneous adipose tissue (SAT) in active acromegaly patients before and after disease control found that 743 differentially expressed genes (DEGs), including 273 down-regulated DEGs and 470 up-regulated DEGs, and those up-regulated DEGs were mainly enriched in fibrosis- and inflammation-related pathways (98). Patients with Laron Syndrome are congenitally deficient in IGF-1. Epidemiological studies have shown that such patients have extremely strong “cancer protection” characteristics (99). Through genome-wide expression profiling analysis of patients, it was found that IGF1 inhibits the expression of TXNIP at the transcriptional level, and TXNIP can be used as a predictive marker for IGF1R-targeted therapy. These findings further reveal the key regulatory role of the GHRH-GH-IGF1 endocrine axis in cancer biology (100).

In addition, transcriptomic studies have also unearthed a series of potential biomarkers. For example, in GH-secreting PitNETs, the expression of the spliceosomal component TCERG1 is significantly higher than that in normal pituitary tissues, and high expression of TCERG1 promotes epithelial-mesenchymal transition (EMT), tumor cell proliferation, and invasion, and reduces the surgical remission rate, suggesting that it might be a biomarker to predict tumor aggressiveness and prognosis (101). Single-cell and spatial transcriptome analyses of GH-secreting PitNETs successfully identified 16 tumor cell subtypes and determined three subtypes associated with malignancy, among which the specific highly expressed transcription factors FOXO1, GTF2IRD1 and MAX were associated with tumor aggressiveness, progression and postoperative hormonal remission rate, respectively, providing a new direction in the development of targeted therapeutic strategies (102).

At the clinical application level, the diagnostic method based on blood transcriptome data combined with machine learning can achieve high-precision diagnosis of GHD in children, and can effectively predict the growth response of GHD patients treated with traditional daily GH therapy or somaplatin, which provides a strong support for the development of preclinical transcriptome-based detection methods and optimization of clinical management (103, 104). Still, transcriptomics alone cannot capture the full complexity of GH proteoforms. Proteoform-centered integration is necessary to connect mRNA signatures to actual functional effectors. By whole transcriptome analysis, GH-secreting PitNETs can be classified into three transcriptome subtypes, among which transcriptome cluster 3 is more invasive, and transcriptome cluster 2 has a smaller tumor size and may be more sensitive to growth inhibitor analogs, which provides an important reference for the development of individualized clinical treatment strategies (105).

However, transcriptome data only reflects the abundance of messenger RNA and cannot fully reflect the complexity of GH proteoforms, because post-transcriptional modifications and protein processing will change the final protein products (71). Therefore, multi-omics integration centered on proteoforms is required to link mRNA expression characteristics with functional proteoforms.

### 4.3 Associations of proteomic variations with GHPs landscape changes in GH-related diseases

Proteomics is promising in clinical applications, enabling disease diagnosis, prognostic assessment, and treatment regimen selection by analyzing body fluids and tissue samples, and mining potential biomarkers (106). Rapid advances have been made in proteomic analysis and identification technologies, the era of proteomics-driven precision medicine is coming (107). Analysis of serum samples from active acromegaly patients before and after disease control revealed that the levels of several proteins were significantly elevated after disease control, and their value as biomarkers of disease activity in acromegaly deserves in-depth study (98). In GNAS mutant tumors, the G protein-coupled receptor (GPCR) pathway is involved in the regulation of tumor endocrine characteristics and therapeutic response, among which ATP2A2 and ARID5B were significantly associated with the rate of change of GH in the octreotide loading assay, and WWC3, SERINC1, and ZFAND3 were correlated with the rate of change of tumor volume after SSA treatment, which are expected to be the molecules that can be used as biomarkers for predicting somatostatin analog (SSA) therapy biomarkers for predicting the effect of SSA treatment (96). In addition, it has been demonstrated that LCN2 expression is upregulated in the serum of children with idiopathic short stature (ISS), which not only inhibits food intake, but also impairs chondrocyte proliferation and growth plate bone formation. LCN2 has a high sensitivity and specificity in distinguishing children with ISS from other children, and may be a useful biomarker for the diagnosis of ISS, as well as a potential therapeutic target (108). Comparative proteomic analysis of sparsely granulated (SG) and densely granulated (DG) GH adenomas identified two key differential proteins, cadherin-1 (CDH1) and catenin beta-1 (CTNNB1). In SG adenomas, proteins related to cell adhesion and extracellular matrix (ECM) are generally down-regulated, while proteins related to DNA replication are up-regulated, which is in line with the clinical features of SG adenomas that are more invasive and have high proliferative activity, and provide potential molecular targets for their diagnosis and treatment (109). In GH-secreting PitNET tissues, our research team has successfully identified 46 GHPs, which is significantly more than the 35 GHPs in the control group. The 11 additional GHPs in GH-secreting PitNETs may provide new molecular clues for mechanistic studies of this disease. Since GH-secreting PitNETs typically lead to acromegaly, these differential proteins may be involved in the onset and progression of acromegaly (36). However, the clinical utility of these GHPs as diagnostic or prognostic biomarkers has not yet been validated in large and independent cohorts, and prospective studies are needed before translation into clinical practice. GH signaling is also shaped by PTMs such as phosphorylation, acetylation, ubiquitination, and glycosylation (36). The influence of these on the occurrence of PitNETs and GH secretion will become a new direction for the mechanism of GH-related diseases.

Despite these advances, the conventional proteomic studies still operate at the peptide level rather than the intact proteoform level, potentially missing critical information about combinatorial PTMs and isoform-specific functions. Furthermore, the reproducibility and clinical validity of these candidate biomarkers must be demonstrated in large, independent cohorts before clinical implementation. Proteoforomics that operate at the proteoform level directly benefit the precise personalized management of GH-related diseases.

### 4.4 Associations of metabolomic variations with GHPs landscape changes in GH-related diseases

Metabolomics has uncovered metabolic fingerprints that are specific to different growth disorders. For the metabolic characterization study of prepubertal and adolescent GHD and ISS, specific metabolic marker combinations were screened and diagnostic models were constructed, which provided a new basis for early differential diagnosis of these diseases (110). In addition, serum metabolite analysis showed that seven metabolites could effectively differentiate between children with normal height, ISS and GHD, and pathways such as sphingolipid signaling pathway, purine metabolism, and sphingolipid metabolism were significantly enriched in children with short stature (111).

Metabolomics data may also provide objective indicators for individualized response prediction to GH therapy. The study on polyethylene glycol recombinant human GH (PEG-rhGH) to treat GHD in children found a significant difference in serum metabolomes between high clinical efficacy group and low clinical efficacy group, and the eight differential metabolites mainly participated in energy metabolism and fatty acid metabolism pathways (112). Since metabolic abnormalities often appear earlier than clinical symptoms, metabolomics has shown exploratory value in the early warning of this type of disease.

In children with GHD, plasma and serum levels of certain ketone bodies, valine, creatine, and other metabolites are elevated, and glutamine levels are decreased, involving multiple metabolic pathways such as ketogenesis and branched-chain amino acid metabolism (113). In AGHD patients, a total of 103 differential metabolites were identified, involving unsaturated fatty acid biosynthesis, sphingolipid metabolism, and other pathways, and recombinant human GH (rhGH) treatment modulated the levels of some glycerophospholipids and fatty acid ester compounds (114). Studies in children with GHD-induced dwarfism have shown that there are mainly disorders of glucose metabolism as well as abnormalities in amino acid metabolism and biosynthesis, and 12 metabolites can be used as potential biomarkers, according to which dietary intervention strategies such as supplementation with betaine, improvement of glucose metabolism, and supplementation with lysine and glutamine have been proposed (115). In patients with acromegaly, 109 different metabolites were identified, involving amino acid derivatives, lipids and bile acid metabolism, which are expected to become new biomarkers and provide new ideas for clinical diagnosis and treatment (116).

There’s growing evidence that systemic metabolic and microenvironmental factors can reshape protein modification landscapes. These factors include hypoxia, oncometabolite-driven metabolic reprogramming, and chronic inflammation, and the systems-level approaches are proposed to link oxidative stress, inflammation, and endocrine complexity (117). These interactive pathways further modulate the dynamic profiles of GHPs, ultimately participating in the pathogenesis of GH-related endocrine and metabolic diseases. For example, hypoxia-inducible factor (HIF)-driven signaling and oncometabolite-regulated exosomes reprogram immune-metabolic crosstalk in type 2 diabetes, atherosclerosis, and cancer (118). Hypoxia also alters cellular redox status. It induces oxidative post-translational modifications like S-nitrosylation and S-glutathionylation (119). Exosomes can transfer metabolic and signaling cargos between cells, influencing metabolic reprogramming in recipient tissues across multiple disease contexts (120). Oncometabolites directly regulate PTM circuits through feedback loops involving metabolite–enzyme interactions. Put all this together, these systemic modifiers may alter the GH proteoform pattern by changing cellular redox status, epigenetic marks, and vesicle-mediated communication (121). Recognizing these interactions is essential to get a holistic view of GHPs dynamics in metabolic and neoplastic diseases.

**Figure 4:**
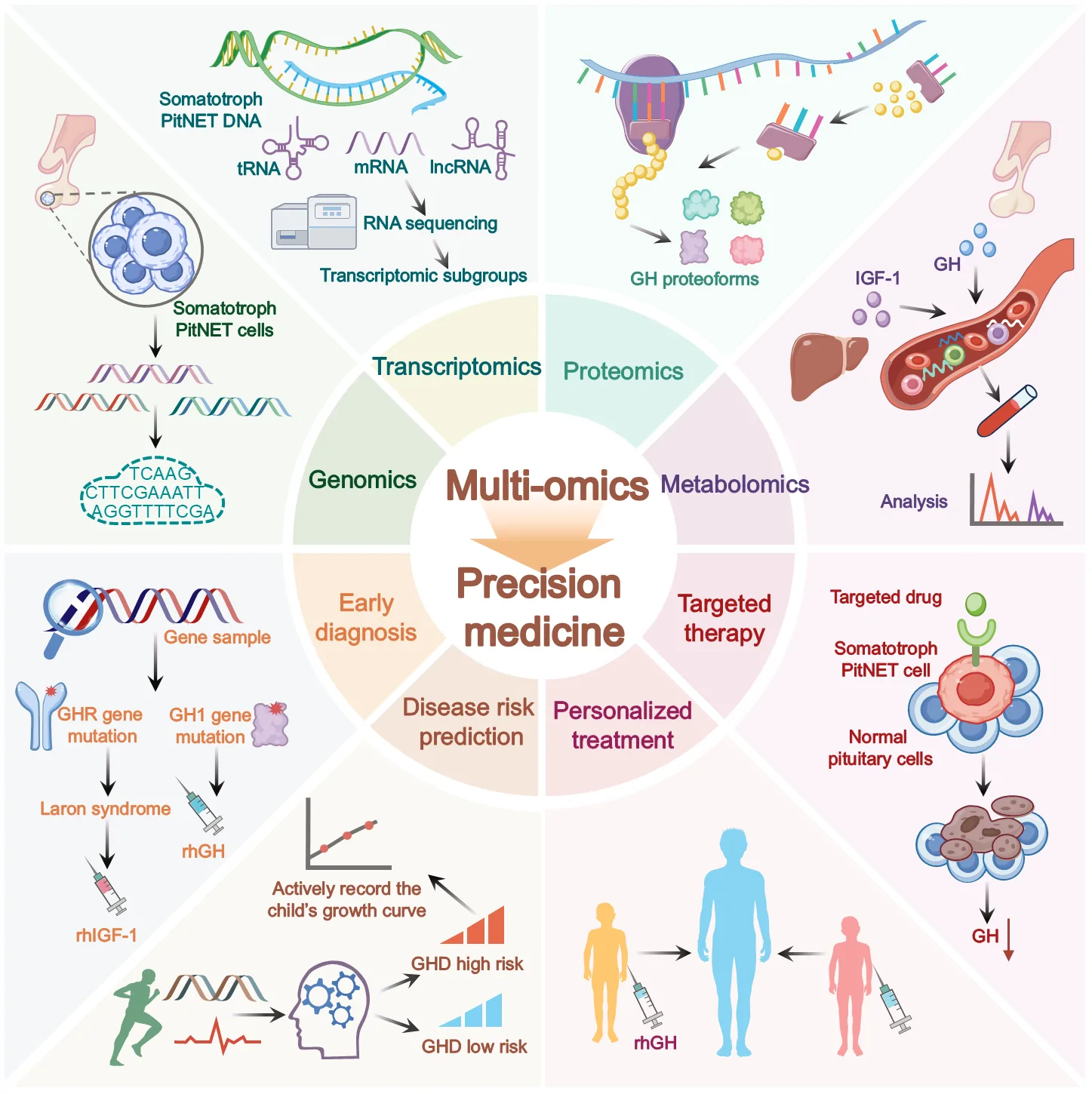
Proteoform-centered multiomics promotes the development of precision medicine in GH-related diseases. Genomic, transcriptomic, proteomic, metabolomic and proteoformic data paint a comprehensive landscape of GHPs, thereby advancing early diagnosis, disease risk prediction, personalized treatment and targeted therapy, and ultimately realizing precision medicine. GH, growth hormone; GHD, growth hormone deficiency; GHR, growth hormone receptor; IGF-1, insulin-like growth factor 1; PitNET, pituitary neuroendocrine tumor; rhGH, recombinant human growth hormone; rhIGF-1, recombinant human insulin-like growth factor 1.

Going forward, future studies should integrate metabolomics with proteoform-resolved proteomics to disentangle these complex regulatory loops. Overall, multiomics data integration has brought new hope for early diagnosis, disease risk prediction, precise drug administration and targeted therapy of GH-related diseases. It’s pushed the diagnostic and treatment model from “one-size-fits-all” toward “individualized” (**Figure 4**). But many challenges remain, such as standardization of multiomics data, optimization of bioinformatics tools, and clinical translation ethics, which need to be further studied and explored. Critically, robust validation of proteoform-based biomarkers in prospective clinical trials remains an unmet prerequisite.

## 5 Therapeutic targets/drugs based on GHPs

### 5.1 The molecular regulatory mechanism of hGHPs

The expression and function of hGHP are regulated at multiple levels: DNA methylation and histone modifications regulate transcription initiation through epigenetic mechanisms, mRNA modifications and non-coding RNA networks finely regulate the processing and translation efficiency of the transcript, and protein structure modifications directly determine its biological activity. The synergy of these mechanisms ensures that hGHP performs precise functions in physiological processes such as growth, development, and metabolic regulation, and abnormal modifications may lead to pathological conditions. Through research at the molecular level hGHP, we can develop targeted drugs to specifically treat GH-related diseases.

For example, DNA methylation and histone modifications dynamically regulate the expression and function of GH gene. The methylation modification of adding a methyl group at the 5th position of the cytosine residue in the hGH gene can inactivate the gene before or at the beginning of gene methylation, and can also directly inhibit transcription by interfering with the binding of transcription factors to the site-specific methylation within the recognition site of the factor (122). During the modification of histones, the methylation, acetylation (123, 124), ubiquitination (125, 126), and phosphorylation (127) at specific sites are also involved to affect the expression of related genes. When transcription factors are embedded in the protein-protein interaction (PPI) network, they can coordinate activities with other transcriptional factors (TFs), chromatin remodeling factors, and auxiliary factors at the enhancer or promoter, regulating the transcription reaction at the precise time window of specific cell types (128). Aberrant promoter hypermethylation in somatotroph adenomas does not completely silence the gene (129). Instead, it leads to massive accumulation of abnormal GH isoforms (59). Demethylating drugs could be developed to reverse this epigenetic abnormality and curb the over-secretion of tumor-specific abnormal GHPs. mRNA modification, as the core content of epigenetic transcriptomics, plays a key role in gene expression regulation by regulating multiple links in the RNA life cycle, and its modification process runs through the entire process from transcription to translation. For example, m6A, m5C, m1A, etc. can be co-transcribedally installed on the nascent transcript by recruiting modification enzymes (130); the typical 5’ cap structure (m7G) enhances mRNA stability and translation efficiency by protecting the 5’ end from degradation by exonucleases and binding to the eukaryotic initiation factor eIF4E (131). Isolated growth hormone deficiency type II (IGHD II) is caused by splice site mutations that result in exon 3 skipping during splicing, leading to massive production of the 17.5 kDa GH isoform and an elevated pituitary 17.5 kDa/22 kDa ratio. The 17.5 kDa isoform forms oligomers with the 22 kDa GH in the endoplasmic reticulum (ER), sequestering the 22 kDa growth hormone within the ER and triggering growth failure. Additionally, the 17.5 kDa isoform exerts toxic effects on the pituitary gland, which may ultimately lead to multiple other hormonal deficiencies (132). Targeting tools such as siRNA or shRNA can degrade transcripts encoding the 17.5 kDa mutant isoform, while histone deacetylase inhibitors (HDACis) modulate splicing by upregulating the splicing factor ASF/SF2 to increase levels of 22-kDa GH and rescue growth impairment (133). In the molecule level of hGHP, disulfide bonds are core elements maintaining the native spatial structure of GH. The disulfide bond is still retained with approximately 60% growth-promoting activity after reduction and S-ureido-methylation of the disulfide bond; reduction and re-oxidation of the hormone can maintain all biological activity (134). Mutations in coding sequences directly disrupt intramolecular disulfide bonds and trigger the production of pathological protein proteoforms. The GH1 c.705G>C mutation converts cysteine at position 53 to serine, abolishing the intramolecular Cys53–Cys165 disulfide bond. Mutant molecules instead form intermolecular disulfide bonds with one another and extensively assemble into inactive dimers within pituitary cells. These mutant dimers nearly lose all growth-promoting activity, which serves as the primary cause of short stature in affected patients (135). In addition, the double basic sequence (Arg167-Lys168) may make the deamidation action a target for proteases, changing the folding of the peptide chain and exposing new regions. The nitration reaction of tyrosine residues and the oxidation reaction of methionine residues also affect the normal physiological function of hGHP (134). These modifications that affect hGH secretion and proteoform generation may become specific targets for targeted therapy of related diseases.

### 5.2 Research progress in GHP-targeted therapy

Regarding the regulatory process of GHPs, GHPs and their signaling pathways have become the key for developing targeted treatments for diseases such as acromegaly and GH deficiency. Currently, many drugs can be used for specific treatment (**Table 1**). Acromegaly is mainly caused by excessive secretion of GH due to PitNETs that secrete GH (165). Surgery is the preferred treatment for acromegaly. For those who refuse surgery, are unsuitable for surgery, or whose condition is not cured by surgery, drug treatment can be chosen. The drugs used to treat acromegaly mainly include SRLs, GHR antagonists, and dopamine agonists (166).

**Table 1:** The drug treatment strategies for GH-related disorders.

Drugs	Diseases	Molecular mechanism	Biological effect	Research models	References
Octreotide LAR Lanreotide depot	Acromegaly (FDA approved)	Affinity for SSTR2 and SSTR5	Used as a first-line drug	Acromegalic patients	(136, 137)
Pasireotide LAR	Acromegaly (FDA approved)	Higher affinity for SSTR5	Among fg-SRL-resistant patients, half may benefit, but worsening glycemic control is a concern	Acromegalic patients	(138)
Oral Octreotide	Acromegaly (FDA approved)	Utilizing transient permeation enhancers to reversibly open tight junctions between intestinal cells.	Effective control of IGF-1 and GH with a safety profile consistent with the approved SRL.	Acromegalic patients	(139)
CAM2029	Acromegaly (FDA not approved)	A novel octreotide formulation employing FluidCrystal technology enables room-temperature storage and self-administration.	The efficacy was significantly better than placebo and was safely tolerated.	Acromegalic patients	(140)
DP1038	Acromegaly (FDA not approved)	Non-invasive intranasal octreotide acetate formulation	Exhibits injection-comparable PK with therapeutic drug concentrations.	Healthy volunteers	(141)
Octreotide implant	Acromegaly (FDA not approved)	Hydrogel-encapsulated for controlled release with six-monthly dosing	Effective maintenance of GH and IGF-1 levels with a favorable safety profile and reduced injection frequency.	Acromegalic patients	(142)
Paltusotine	Acromegaly (FDA not approved)	SSTR2 selective agonist	Can be administered orally, 70% bioavailability of oral solution, 4000 times more selective for SSTR2 than other receptor subtypes.	Acromegalic patients	(143)
ONO-5788	Acromegaly (FDA not approved)	An SSTR2 selective agonist and its metabolite ONO-ST1–641 also signals through SSTR2	Significantly inhibited GHRH-induced GH secretion and basal GH secretion	Rats	(144)
ONO-ST-468	Acromegaly (FDA not approved)	SSTR2 selective agonist	Similar GH inhibition to octreotide	Male cynomolgus monkeys	(145)
Somatoprim (DG3173, PTR-3173)	Acromegaly (FDA not approved)	Has an affinity for SSTR2, SSTR4, SSTR5	Effectively inhibits GH secretion and has very low inhibitory activity on insulin secretion.	GH-secreting PitNET Sample	(146)
AP102	Acromegaly (FDA not approved)	Has a balanced affinity for SSTR2 and SSTR5	Comparable or superior to existing therapies in inhibiting hormone secretion and tumor proliferation without risk of hyperglycemia	HEK293 cell lines transfected with hSSTR2 and hSSTR5	(147)
GT-02037	Acromegaly (FDA not approved)	SSTR1~5 agonists	A glycosylated growth inhibitor with affinity for five receptors.	NR	(148)
TBR-065 (BIM-23B 065)	Acromegaly (FDA not approved)	Second-generation SSTR-DA chimeras	Significantly better than existing therapies in inhibiting GH secretion, and its metabolites do not affect its own activity	GH-secreting PitNET Sample	(149)
Pegvisomant	Acromegaly (FDA approved)	GHR antagonist	Treatment with monotherapy or in combination with SRL improves the patient’s condition	Acromegalic patients	(150)
S1H	Acromegaly (FDA not approved)	GHR antagonist	Inhibiting HGH-induced STAT5 phosphorylation in a dose-dependent manner has a stronger antagonistic effect on hGHR than pegvisomant and is highly	Cell	(151)
AZP-3813	Acromegaly (FDA not approved)	GHR antagonist	Effectively reduce the levels of IGF-I in rats and Beagle dogs in a dose-dependent manner.	Rats Beagle dogs	(152)
ATL1103	Acromegaly (FDA not approved)	Antisense oligonucleotides of GHR	Reduced GHR expression and significantly lowered IGF-1 levels in patients.	Acromegalic patients	(153)
ISIS 766720	Acromegaly (FDA not approved)	Antisense oligonucleotides of GHR	It led to a significant decrease in the AUC of GHBP and IGF-1, while the GH level did not increase, with good safety and tolerance	Acromegalic patients	(154)
Cabergoline	Acromegaly (FDA not approved)	Dopamine Agonists	Monotherapy normalized IGF-1 in 1/3 of patients, and combination therapy normalized IGF-1 when SRL was ineffective	Acromegalic patients	(155)
Raf Kinase Inhibitory Protein	Acromegaly (FDA not approved)	Non-phosphorylated RKIP attenuates MAPK signaling through binding and inhibition of Raf1 kinase	Low levels of RKIP predict poor patient response to octreotide therapy.	Acromegalic patients	(156)
rhGH	Noonan; PGHD; AGHD; ISS; Turner; SHOX deficiency; Prader-Willi syndrome in children; SGA; Chronic Kidney Disease in Children; HIV Adult-associated weight loss or wasting; iPPSD2/PHP1A (Therapeutic iPPSD2/PHP1A not approved, rest approved by FDA)	Similar to endogenous GH, acts mainly through IGF-1 mediation	It improved the height issue of children, increased muscle mass and weight for adults, and enhanced mental health and physical function.	Patients diagnosed with the above diseases	(157, 158)
rhIGF-1	Laron (FDA approved)	Directly replacing the physiological function of endogenous IGF-1	After long-term treatment, the patient’s height improved considerably.	Patients with Laron syndrome	(159)
somapacitan	GHD, AGHD (FDA approved)	Reversibly binds endogenous albumin to prolong its action *in vivo*	LAGH,weekly efficacy and safety similar to once-daily GH	PGHD patients AGHD patients	(160)
Ngenla (somatrogon)	PGHD (FDA approved)	Glycosylation modifications and CTP structural domains prolong the duration of action	LAGH, weekly dosing	PGHD patients	(161)
Skytrofa (lonapegsomatropin)	PGHD (FDA approved)	Prolongation of action by physical shielding of the mPEG carriers	LAGH, weekly dosing	PGHD patients	(162)
LUM-201	PGHD (FDA not approved)	Enhancement of endogenous GH secretion through activation of GHSR1a	GH-promoting effect was significantly better than that of standard stimulants, and the difference in response correlated with baseline IGF-1	PGHD patients	(163)
Tesamorelin	HIV-associated lipodystrophy syndrome (FDA approved)	Binding to GHRHR activates the AC-cAMP-PKA signaling pathway and promotes the synthesis and secretion of GH.	Selective reduction of visceral fat, preservation of subcutaneous fat, improvement of body image, good safety.	Patients with HIV-associated lipodystrophy syndrome	(164)

The current drug recommendation schemes are based on the “trial and error” method. Octreotide and Lanreotide in the first-generation SRLs are the first-line drugs for treating acromegaly patients, inhibiting GH release by binding to somatostatin receptor 2 (SSTR2) and somatostatin receptor 5 (SSTR5) (167). Monotherapy or combination therapy with other drugs is effective. The new drugs AP102 (147) and TBR-065 (149) further optimize this mechanism by balancing receptor affinity or enhancing efficacy through dopamine receptor activity. Postoperative tumor remnants, sparse granular GH-secreting tumors, high GH and IGF-1 secretion, and high proliferation index of the tumor often indicate that patients are resistant to first-generation SRLs (72). When first-generation SRLs fail to achieve disease control, second-line treatment is required. The second-generation SRL Pasireotide is a second-line drug for treating acromegaly, with a higher affinity for SSTR5 (168). The α cells of glucagon mainly express SSTR2, and the β cells of insulin mainly express SSTR2 and SSTR5. After using this drug, insulin secretion significantly decreases, only moderately inhibiting glucagon secretion, and patients may experience elevated blood sugar levels, and diabetic patients may have problems with worsening blood sugar (168).

**Figure 5:**
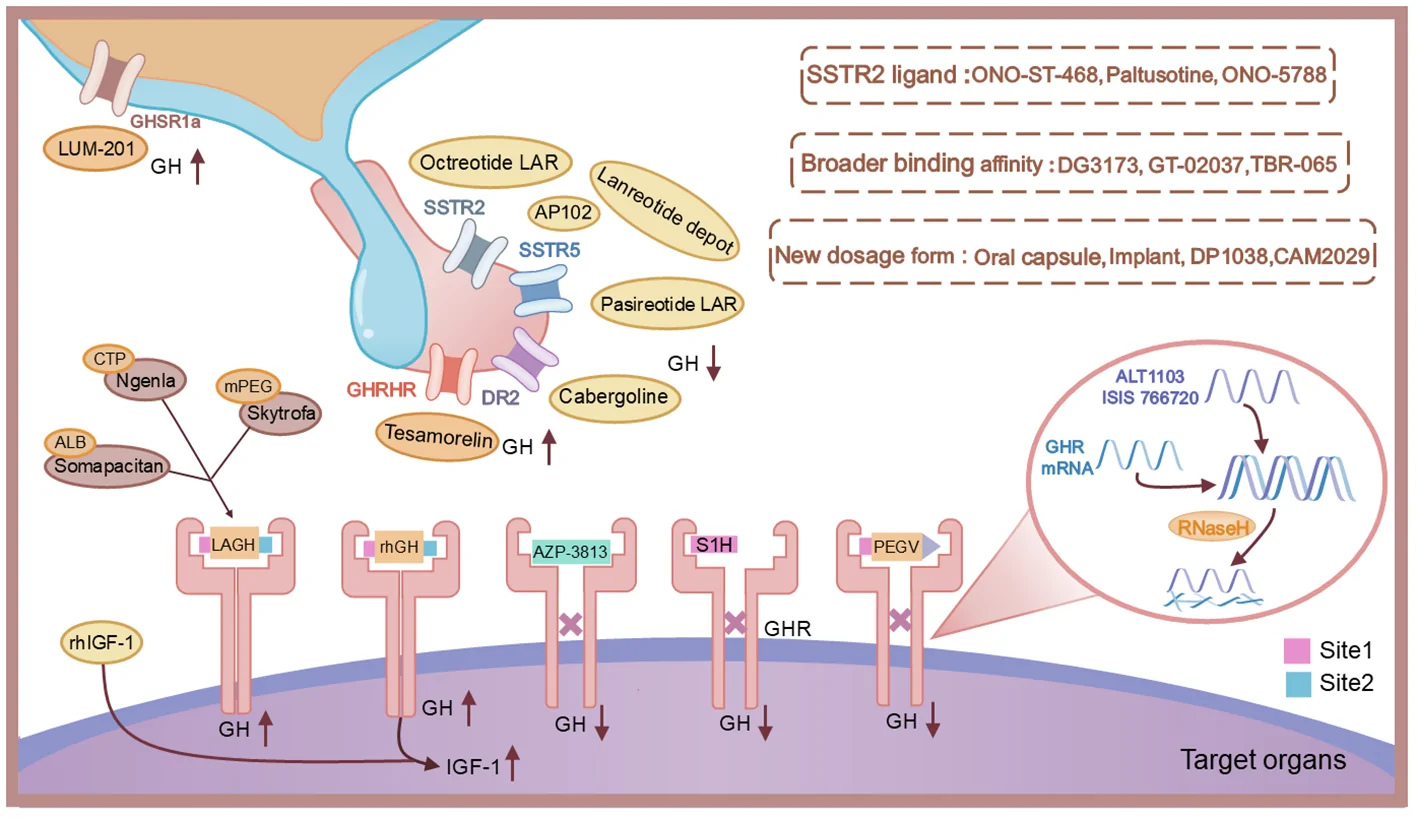
Therapeutic landscape of GH-related disorders. This image illustrates the current drugs used for the treatment of GH-related diseases as well as their action targets. These drugs mainly bind to specific receptors on the pituitary and target organs, inhibiting excessive GH secretion or ameliorating insufficient secretion through different mechanisms. In addition, several novel drugs currently under development are also listed in the image. ALB, albumin; CTP, C-terminal peptide; DR2, dopamine receptor 2; GHR, growth hormone receptor; GHRHR, growth hormone-releasing hormone receptor; GHSR1a, growth hormone secretagogue receptor type 1a; IGF-1, insulin-like growth factor 1; LAGH, long-acting growth hormone; LAR, long-acting repeatable; mPEG, methoxypolyethylene glycol; PEGV, pegvisomant. rhGH; recombinant human growth hormone, rhIGF-1; recombinant human insulin-like growth factor 1, S1H; site 1-binding helix; SSTR2, somatostatin receptor 2; SSTR5, somatostatin receptor 5.

Pegvisomant is currently the only approved growth hormone receptor (GHR) antagonist, suitable for second-line or third-line treatment (169). Pegvisomant binds to the first GHR monomer through site 1, but site 2 is mutated and thus unable to effectively bind to the second GHR monomer, preventing downstream signal trans duction. Pegvisomant can restore IGF-I levels in 58% to 97% of patients, with a high response rate (170). The development of novel GHR antagonists is steadily advancing, with site 1-binding helix (S1H) and AZP-3813 being two important compounds currently under investigation. S1H competitively occupies the site 1 binding domain of GHR by mimicking the mini-helix structure of site 1 of GHP, thereby preventing specific GHP from binding to the receptor and blocking its signal transduction (151). AZP-3813 is a 16-amino acid bicyclic peptide that has an affinity for GHR and blocks the binding of GHPs to GHR (152). When choosing a second-line treatment drug, sparse granular GH-secreting tumors, low AIP expression, SSTR5 cytoplasmic expression, and carrying flfl-GHR heterozygotes can consider Pasireotide Lar. For patients with low cytoplasmic expression of SSTR2 and low or low expression of SSTR5 and carrying d3-GHR heterozygotes, pegvisomant can be considered (139) (**Figure 5**). Cabergoline is a long-acting dopamine receptor 2 (DR2) selective agonist, with little effect on hormone relief, mainly used for patients with mild conditions after pituitary surgery (171). The long-term SRL administration route is monthly intramuscular injection, requiring assistance from medical staff, and injection sites may experience swelling, pain, or bruising, and patients may have increased psychological burden (172). Various new formulations have been developed, such as oral capsules, nasal preparations, and sustained-release implants, to improve patient compliance. ATL1103 and ISIS 766720 are antisense oligonucleotides targeting GHR, inhibiting the translation of GHR mRNA, effectively reducing IGF-I levels in patients with acromegaly, and are currently in phase 2 clinical trials (166). Non-phosphorylated Raf kinase inhibitory protein (RKIP) inhibits Raf1 kinase by binding and suppressing, weakening MAPK signal transduction, and low-level RKIP indicates a poor response to octreotide treatment, which may be a potential therapeutic target in the future (156). Also, several selective SSTR2 agonists and broad-spectrum SSTR agonists capable of targeting multiple subtypes, which are currently under development, have demonstrated significant therapeutic potential.

But here’s an important reality check that these therapies are at very different stages of readiness. First-generation SRLs and pegvisomant are established, whereas antisense oligonucleotides are still early-stage. And oral nonpeptide SSTR2 agonists have recently been approved (173). To date, multiple GHPs have been found in PitNETs, but whether any of these therapies were actually designed to target specific proteoforms that’s still an open question (36, 72).

Looking forward from a proteoform-centric view, future drug development should aim to selectively inhibit pathogenic GHPs while preserving the functions of beneficial ones (63, 71, 165, 174–176). That would require proteoform-resolved pharmacodynamics and the establishment of regulatory frameworks to demonstrate clinical utility of proteoform-based stratification. For example, GHPs that exhibit aberrant receptor binding or sustained signaling activity can lead to the development of specific GHR antagonist, which can inhibit specific GH species rather than universally inhibits all GH species. While proteoform-informed drug design is a promising research direction, it should be pursued in parallel with, not as a replacement for, established therapeutic strategies. Through table classification, sorting out the types of relevant diseases, the current mainstream clinical intervention schemes, and the obstacles encountered in the clinical application of various intervention measures, it intuitively reflects the core shortcomings of the existing therapies that lack subtype selectivity, and highlights the research and development value of layered targeted new drugs (**Table 2**).

**Table 2:** Growth hormone-regulated diseases, clinical intervention methods, and reasons for intervention limitations.

Disease/state	GH isoform abnormal indicators	Clinical interventions	Limitations	References
Active acromegaly	The ratio of non-22 kDa/20 kDa GH is increased; high non-22 kDa GH represents post-operation residual tumor	Pituitary adenoma surgery + octreotide	Octreotide preferentially inhibits 22 kDa GH; residual tumor secretes abnormal GH; no isoform-targeted drugs	(39, 176, 177)
Adult GHD	20 kDa and 22 kDa GH are both decreased; the ratio of 20 kDa/22 kDa GH is no changed relative to healthy control	Subcutaneous 22 kDa rhGH replacement	Exogenous 22 kDa GH suppresses endogenous 20 kDa GH; disrupts natural isoform balance	(176, 177)
Anorexia nervosa	20 kDa GH ratio vs healthy is increased	Nutritional + psychological rehab	No drug adjusts abnormal 20 kDa GH proportion	(176)
IGHD type I	GH1 deletion/nonsense causes near complete loss of functional GH isoforms	Early rhGH (higher dose than II); GnRH-a for puberty	High anti-GH antibody risk; late diagnosis causes worse final height	(178, 179)
IGHD type II	GH1 splicing mutation causes toxic 17.5 kDa GH excess; higher expression represents worse phenotype	22 kDa rhGH; GnRH-a during puberty	RhGH cannot eliminate toxic 17.5 kDa GH or reverse pituitary hypoplasia; late diagnosis causes worse height gain; siRNA is verified only *in vitro* to specifically degrade toxic 17.5 kDa GH mRNA	(178, 180)

For GHD, rhGH is used for various indications such as pediatric GHD, adult GHD, HIV-related weight loss or emaciation in adults, etc. It improves height, muscle mass, and physical function through IGF-1 mediation. When it comes to height improvement, rhGH is administered subcutaneously once daily, but patient compliance is poor (181). Therefore, various long-acting GH (LAGH) preparations have been developed to achieve weekly administration, with similar efficacy and safety as the daily administration preparations, significantly enhancing patient compliance. Clinicians should screen patients suitable for LAGH treatment and master the methods of treatment monitoring and dose adjustment. In addition, long-term studies should be conducted to evaluate the compliance of medication, clinical efficacy, cost-effectiveness, and safety of LAGH preparations. LUM-201 is a GHRH that can be administered orally and is used to treat pediatric GHD, aiming to increase the release of endogenous GH and promote pulsatile secretion (182). Some GHD patients may have insufficient sensitivity to LUM-201 due to differences in the remaining hypothalamic-pituitary function. Based on this, the “baseline IGF-I concentration > 30 ng/mL” and “single dose LUM-201 GH peak response ≥ 5 ng/mL” two indicators were integrated to establish a predictive enrichment marker (PEM) model. A positive PEM can choose LUM-201 treatment, while a negative PEM is more suitable for rhGH injection therapy. Although precisely differentiating different pathological characteristics of patient groups, the treatment selection can be optimized and ineffective medication can be avoided (183). In addition to GHD, GH-related therapies also show significant efficacy in other endocrine metabolic diseases. Recombinant human IGF-1 (rhIGF-1) can provide exogenous IGF-1 and improve the height problem of patients with Laron syndrome (159). Tesamorelin can promote GH synthesis and secretion by binding to GH-releasing hormone receptor (GHRHR), improving fat metabolism disorders related to HIV (164).

Targeted therapy based on GHPs and their dynamic signaling pathway networks provide diverse treatment options for GH-related diseases such as acromegaly and GHD. The optimization of the mechanism of action of traditional drugs, the innovation of new formulation types, and personalized drugs based on proteoforms are advancements that have pointed the way for future research (184). But to realize proteoform-informed therapy, future drug development must incorporate proteoform-resolved pharmacodynamics and tackle the regulatory requirements for showing clinical utility of proteoform-based stratification, which will be further addressed in the section “Future perspectives”.

## 6 Future perspectives

### 6.1 In-depth insights into GH proteoformics in GH-related diseases

GH is a key regulator of human growth, but diagnosis and treatment for GH-related diseases still need work. The concept of proteoformics has shown that different proteoforms from the same GH gene matter a lot in disease pathogenesis. Digging into GHPs heterogeneity can help ones understand how diseases start, predict them, and develop targeted inhibitors. This helps traditional medicine evolve toward predictive, preventive, and personalized medicine (PPPM; 3PM) (1, 36).

#### 6.1.1 GHPs and modifications in GH-related diseases

Under physiological conditions, the pituitary secretes multiple GH isoforms. In GH-related diseases, the GHPs and their modifications in pituitary tissues change significantly. In patients with GHD, whether due to hypothalamic-pituitary dysfunction or hereditary GHD, the synthesis and secretion of GH by pituitary somatotrophs decrease, and the types and proportions of GHPs may also become abnormal (36). Absence or reduction of specific GHPs or GHR defects may fail to activate downstream signaling, affecting normal growth (185). Phosphorylation of some GHPs involved in growth signal transduction could lower receptor affinity and block signal transmission (186).

In GH-secreting PitNETs, changes in GHPs are more complex. Our previous study identified 46 GHPs in GH-secreting PitNETs tissues versus 35 in normal pituitary tissues. Among these, 11 GHPs are unique to PitNETs. Modification-site changes included phosphorylation at Ser77, Ser132, Thr174, and Ser176; ubiquitination at Lys96; acetylation at Lys171; and deamidation at Asn178 (36). These changes could affect GH structure, activity, and receptor-binding ability, thereby disrupting cell growth and affecting tumor development.

In addition, differentially modified GHPs participate in distinct intracellular signaling pathways. Under physiological conditions, specifically modified GHPs bind to their receptors, activating downstream growth signaling pathways to maintain normal cell growth and metabolism. But in disease, abnormally modified GHPs may bind receptors wrongly or turn on incorrect pathways and these changes are closely associated with disease symptoms. Studying GHPs and their modifications in pituitary tissues of GH-related diseases not only helps understand disease mechanisms, but also might provide directions for identifying potential biomarkers and therapeutic targets, advancing the development of predictive diagnosis, targeted prevention, and personalized medical services for these diseases.

Meanwhile, given that GHPs are released into the blood and serum testing features cost-effectiveness, minimal invasiveness and high accuracy. Therefore, serum GHPs represent promising candidate biomarkers, but their sensitivity, specificity, and predictive value require rigorous evaluation in multicenter prospective studies. In terms of disease treatment, therapeutic targeting can be directed not only at GHPs but also at GHRPs. Specific interference with the binding interaction between GHPs and GHRPs is expected to yield therapeutic effects for GH-associated disorders.

#### 6.1.2 Clinical application of GHPs patterns in GH-related diseases

**Figure 6:**
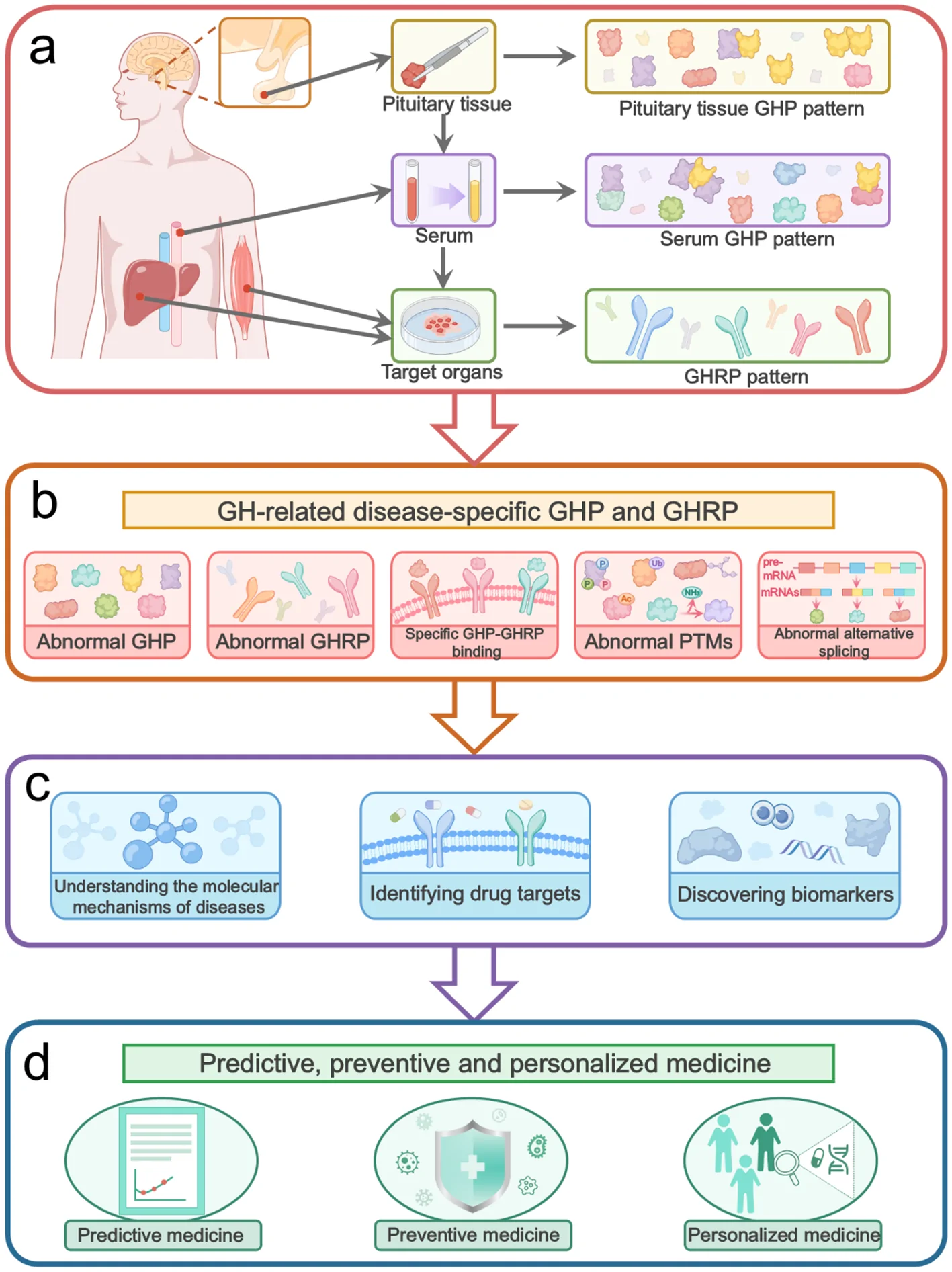
Establish the reference map of GHPs and GHRPs to identify GH-related disease-specific GHPs and GHRPs. **(a)** Specific GHPs and GHRPs were isolated from the pituitary tissues, serum and target organs of patients; **(b)** These GHPs and GHRP include abnormal GHPs and GHRPs, their abnormal binding, abnormal post-translational modifications, and abnormal alternative splicing. The reference maps of GHPs and GHRPs can be established based on these specific GHPs and GHRPs; **(c)** This helps ones understand the molecular mechanisms of diseases, identify drug targets and discover biomarkers; **(d)** Ultimately, it realizes 3PM, namely predictive medicine, preventive medicine and personalized medicine. Ac, acetyl group; GH, growth hormone; GHPs, growth hormone proteoforms; GHRPs, growth hormone receptor proteoforms; PTMs, post-translational modifications; Ub, Ubiquitin.

It is worth noting that the construction of a GHPs reference map and its use for disease screening remain hypothetical (87). Preliminary profiling of GHPs has merely been accomplished using limited pituitary tissue specimens so far, while a standardized population-wide reference atlas for serum GHPs stratified by age, gender and physiological conditions is still unavailable (1). In the clinical diagnosis and assessment of GH-related diseases, molecular biological detection has higher diagnostic sensitivity and timeliness advantages over imaging examinations. Molecular-level detection can identify abnormal situations at the early stage of the disease and has priority value in disease screening and early intervention. And proteoforms remain a largely uncharacterized dimension in biomedical research, implying that the systematic resources required for clinical translation of proteoform biomarkers are still lacking (71). Based on this, we expect to be able to construct a GHPs reference map, and characterize the molecular features of the GH proteoformics through high-resolution mass spectrometry analysis and modeling systems. Existing proteoformics reference databases can be compared with patient proteomics data; by analyzing differential protein/proteoform expression profiles, these databases could enable rapid screening and precise identification of GH-related disorders (**Figure 6**). Currently, there are joint experiments of the molecular profile of tumor DNA and personalized treatment related to the development of the treatment of pituitary adenomas with multiomics tumor profiling, which is also a good beginning (187, 188). Looking ahead, biosensor technologies demonstrate the feasibility of real-time growth hormone detection using cost-effective and portable platforms (189). Meanwhile, AI and machine learning approaches, such as facial image analysis and MRI radiomics, show potential to aid the diagnosis of GH-associated disorders (190, 191). However, integrated platforms pairing GHP real-time monitoring with AI-driven prediction for early disease warning are still theoretical, with no relevant clinical trials or published findings available. Although integrating 3PM with big data models is still early and currently faces challenges like data collection, equipment costs, and cybersecurity, this approach holds exploratory potential for future diagnosis and treatment of human diseases (192).

### 6.2 Integration of GH proteoformics and other omics data for GH-related diseases

**Figure 7:**
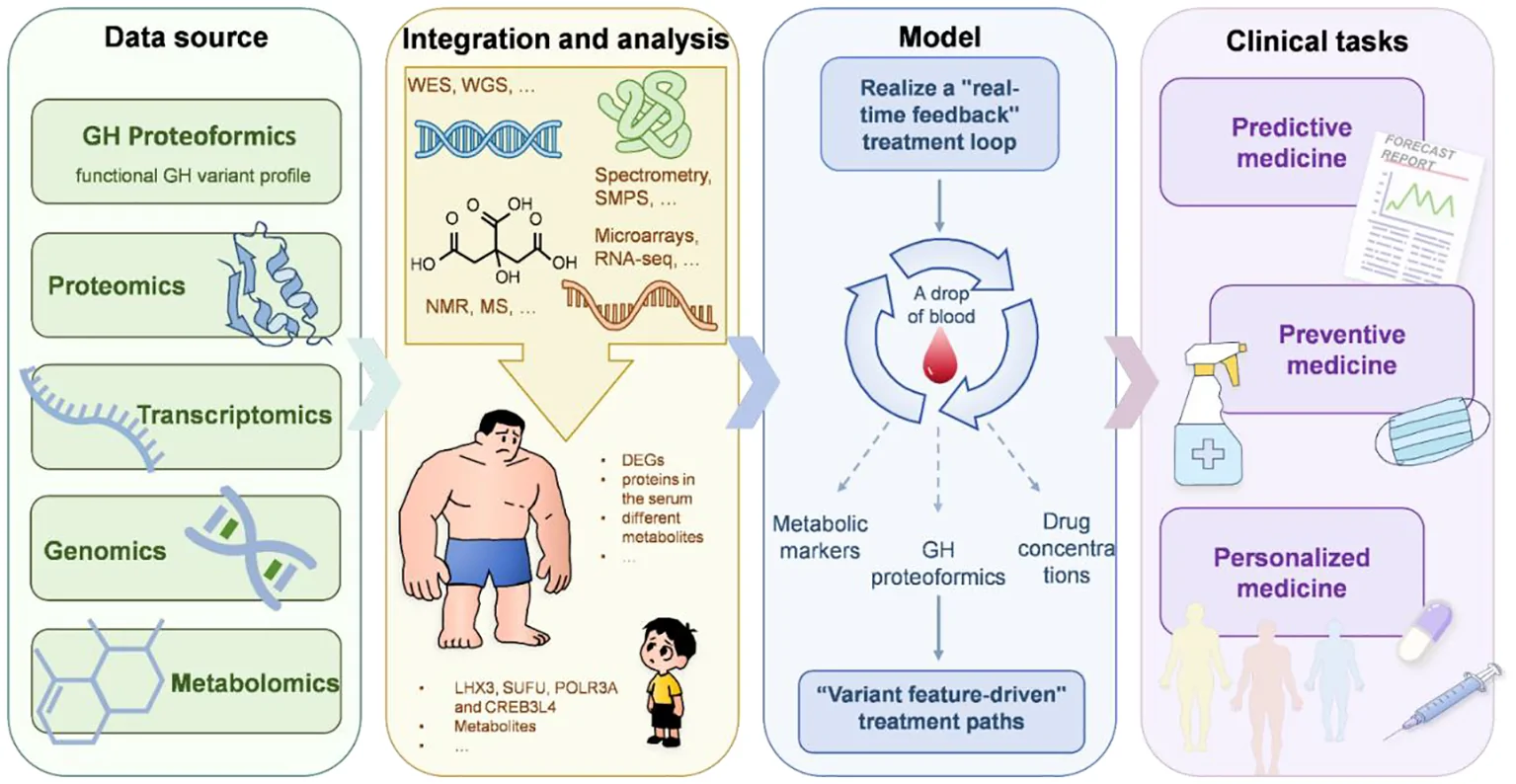
The combination of GH proteoformics and multiomics. GH proteoformics is integrated with multi-dimensional data from genomics, proteomics, transcriptomics and metabolomics. These data undergo integration and analysis using approaches such as WES and RNA-seq, which further facilitate the construction of 'proteoform feature-driven' treatment paths. Ultimately, this framework enables the achievement of 3PM, namely predictive medicine, preventive medicine, and personalized medicine. GH, growth hormone; MS, mass spectrometry; NMR, nuclear magnetic resonance; SMPS, single-molecule protein sequencing; WES, whole-exome sequencing; WGS, whole-genome sequencing.

Optimizing long-acting GH inhibitors, exploring non-injection routes, and developing drug delivery strategies for GH-related diseases proteins warrant further in-depth research. Through genomic, metabolomic, and proteomic analyses, identifying specific targets, developing targeted drugs, and formulating precise dosing plans can help reduce over-treatment and ineffective intervention (193). The core value of GH proteoformics lies in revealing the “functional GHP profile”—that is, the association between different GHPs and disease phenotypes. When this information is integrated with genomics, transcriptomics, and metabolomics, it can form a multi-dimensional therapeutic target network of “proteoform-gene-metabolism” (**Figure 7**). Currently, MS technology has played a significant role in therapeutic drug monitoring (TDM), pharmacoepigenomics, pharmacomicrobiomics, pharmacogenomics (PGx), and immunopeptidomics. These five fields are crucial for individualized treatment of patients (194). In the future, we hope that these technologies can be widely applied in the treatment of various diseases, and the integration of innovative drug delivery technology with multiomics is emerging as a promising trend (193).

This integration also provides a molecular basis for optimizing non-injectable administration routes. For oral or transdermal delivery, the stability of GHPs and their metabolic characteristics in the body need to be considered to avoid premature or delayed degradation of the drug. Meanwhile, by using single-cell transcriptomics to identify surface markers of cells with high GHRP expression in target organs, targeted delivery of carriers can be achieved, reducing systemic exposure. In terms of precise dosing, the combination of GH proteoformics and TDM will revolutionize the traditional “body weight/body surface area” dosing model. Through real-time monitoring of the proportion of various GHPs in serum using mass spectrometry, combined with the patient’s GH gene splicing subtypes, the administration frequency and dosage of recombinant GH can be dynamically adjusted to avoid ineffective medication.

Looking forward, as MS is gradually applied in clinical laboratories, the integration of GH proteoformics with multiomics data is expected to enable a closed-loop therapeutic system with “real-time feedback”: after treatment, a single drop of blood would simultaneously detect the GHP profile, metabolic markers, and drug concentrations. Combined with pre-set multiomics models, treatment plans might support the dynamic adjustment. This model will not only minimize over-treatment but also customize “proteoform feature-driven” treatment paths for each type of GH-related disease, ultimately achieving a leap from “symptom-based treatment” to “cause-based regulation”. Designing drugs via proteoformics to selectively clear pathogenic proteoforms while retaining normal ones is another key step toward precision endocrinology (73).

Despite the promise of proteoform-based diagnostics and therapeutics, substantial work is needed before they reach the clinic (63, 71). First, validation and reproducibility: prospective, multicenter cohort studies are urgently required to validate the diagnostic and prognostic performance of candidate GHPs panels against current gold standards (195). Second, standardization: efforts to standardize MS-based proteoform quantification across different platforms and laboratories, including the development of certified reference materials, are a prerequisite for regulatory approval (87). Third, regulatory frameworks: paths for proteoform-based *in vitro* diagnostics and companion diagnostics remain to be clearly defined by agencies like the FDA and NMPA. Addressing these challenges will be essential to translate proteoform-centric discoveries into clinical practice.

## 7 Conclusions

GH is a pivotal regulator of growth, metabolism, and tissue homeostasis, and its study has become a paradigm for translating molecular insights into precision therapeutic strategies. Major advances in proteoformics and multiomics (genomics, transcriptomics, and metabolomics) have unraveled the complexity of GH biology. High-resolution MS and spatial proteoformics have identified disease-specific GHPs and their post-translational modifications (including phosphorylation, acetylation, ubiquitination, and glycosylation), while machine learning models now predict treatment responses. These technologies have also enabled the discovery of biomarkers, refining diagnostic accuracy and therapeutic stratification.

The clinical management of GH-related disorders has evolved from empirical “trial-and-error” approaches toward personalized strategies. In acromegaly, first-line SRLs like octreotide have been complemented by second-line agents, with novel formulations improving compliance. For GHD, weekly long-acting GH formulations have mitigated daily injection burdens, while oral GHRH agonists offer mechanistic alternatives for select patients. Emerging therapies, including antisense oligonucleotides targeting GHR and dopamine receptor agonists, further expand therapeutic landscape. Moreover, systemic pathophysiological modifiers such as hypoxia, oncometabolite-driven metabolic reprogramming, and chronic inflammation may shape GHPs dynamics through redox-sensitive modifications and exosome-mediated crosstalk, an area that warrants integrated multiomic investigation.

Looking forward, the integration of “multiomics-driven precision medicine” with innovative drug delivery technologies promises to redefine GH therapy. However, substantial challenges remain before proteoform-based diagnostics and therapeutics reach clinical practice. These include: (i) prospective, multicenter validation of candidate GHPs panels against current gold standards; (ii) standardization of mass spectrometry-based proteoform quantification across platforms and laboratories, including the development of certified reference materials; and (iii) establishment of regulatory pathways for proteoform-based *in vitro* diagnostics and companion diagnostics by agencies such as the FDA and NMPA. Addressing these hurdles will require collaborative, interdisciplinary efforts.

Importantly, GHPs is not intended to position proteoform analysis as a central solution for diagnosis and therapy. Rather, proteoformics should be viewed as an evolving tool that, when integrated with genomics, transcriptomics, metabolomics, and robust clinical phenotyping, can contribute to a more nuanced understanding of GH biology. The ultimate clinical management of GH-related diseases will continue to rely on established endocrine principles, with proteoform-based approaches serving as potential adjuncts after rigorous validation. By bridging molecular mechanisms with clinical innovation, GH proteoformics research is poised to advance 3PM for endocrine and metabolic disorders, ensuring that future interventions are both mechanistically informed and patient-centric.

## Glossary

AGHD: adult growth hormone deficiency
Akt: Ak strain transforming.
AI: artificial intelligence
CS-A: chorionic somatomammotropin hormone A
CS-B: chorionic
CS-L: chorionic somatomammotropin hormone like 1
DEGs: differentially expressed genes
ERK: extracellular regulated kinase
GH: growth hormone
GHD: growth hormone deficiency
GHI: growth hormone
GHPs: GH proteoforms
GHR: growth hormone receptor
GHRPs: GH receptor proteoforms
GHRH: GH-releasing hormone
hGH: human growth hormone
hGHR: human growth hormone receptor
ISS: idiopathic short stature
IGF-1: insulin-like growth factor 1
IGHD IA: isolated growth hormone deficiency type IA
JAK2: Janus kinase 2
LAGH: long-acting GH
PIP₃: Phosphatidylinositol (3,4,5)-trisphosphate.
PIP₂: Phosphatidylinositol (4,5)-bisphosphate.
PI3K: Phosphoinositide 3-kinase.
pGH: placental growth hormone
PEG-rhGH: polyethylene glycol recombinant human GH
PTMs: post-translational modifications.
PEM: predictive enrichment marker
PTPs: protein tyrosine phosphatases
PEA: proximity extension assay
RKIP: Raf kinase inhibitory protein
Raf: rapidly accelerated fibrosarcoma
rhGH: recombinant human growth hormone
STAT5: signal transducer and activator of transcription 5.;SWS, slow-wave sleep
SST: somatostatin
SSA: somatostatin analog
SSTR2: somatostatin receptor 2
SSTR5: somatostatin receptor 5
SRLs: somatostatin receptor ligands
SG: sparsely granulated
SOCS: suppressor of cytokine signaling
TDM: therapeutic drug monitoring
Trio-WES: trio-whole exome sequencing
2-DE: two-dimensional gel electrophoresis
